# Supplementary material for: Multimodal analysis demonstrating the shaping of functional gradients in the marmoset brain
Source: Nat Commun. 2022 Nov 3;13:6584. doi: 10.1038/s41467-022-34371-w (PMC9633775; doi:10.1038/s41467-022-34371-w)
Supplement: Supplementary file 1 — Supplementary Information [file 41467_2022_34371_MOESM1_ESM.pdf]

Supplementary Information

Title: Multimodal analysis demonstrating the shaping of functional gradients in the marmoset brain

Chuanjun Tong[1,2,4,5], Cirong Liu[2], Kaiwei Zhang [2], Binshi Bo[2], Ying Xia[2], Hao Yang[2], Yanqiu Feng[1,4,5*], Zhifeng Liang[2,3*]

[1] School of Biomedical Engineering, Southern Medical University, Guangzhou, China

[2] Institute of Neuroscience, CAS Key Laboratory of Primate Neurobiology, Center for Excellence in Brain Science and Intelligence Technology, Chinese Academy of Sciences, Shanghai, China

[3] Shanghai Center for Brain Science and Brain-Inspired Intelligence Technology，Shanghai, China.

[4] Guangdong Provincial Key Laboratory of Medical Image Processing & Guangdong Province Engineering Laboratory for Medical Imaging and Diagnostic Technology, Southern Medical University, Guangzhou, China

[5] Guangdong-Hong Kong-Macao Greater Bay Area Center for Brain Science and Brain-Inspired Intelligence & Key Laboratory of Mental Health of the Ministry of Education, Southern Medical University, Guangzhou, China

*Correspondence should be addressed to Yanqiu Feng (foree@smu.edu.cn) or Zhifeng Liang (zliang@ion.ac.cn).

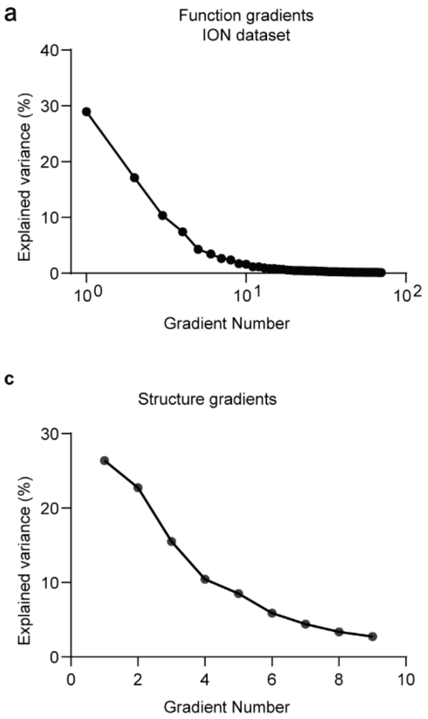

Supplementary Fig. 1 Explained variance accounted by marmoset gradient components.

(a,b) Explained variance accounted by functional gradient components. The first four functional gradients accounted for at least 5 % variance in ION dataset (a) and NIH dataset (b).

(c) Explained variance accounted by structural gradient components. The first four structural gradients accounted for at least 10 % variance.

Source data are provided as a Source Data file.

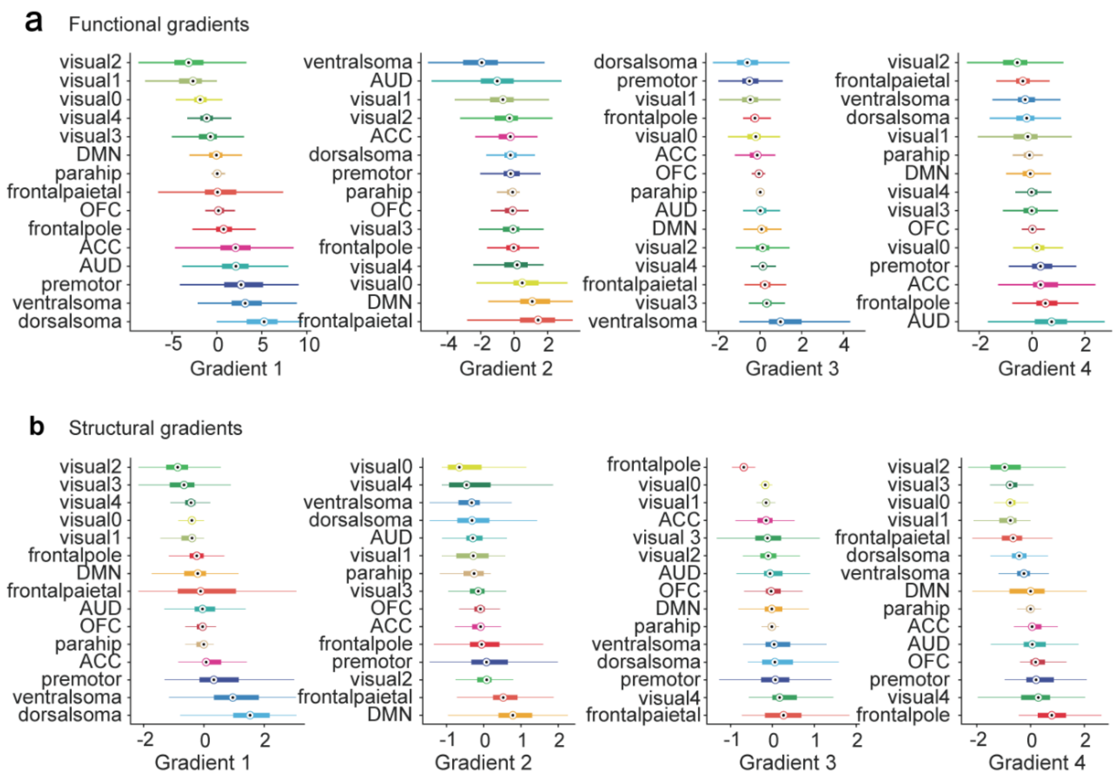

Supplementary Fig. 2 Gradient values across marmoset networks.

Functional (a) and structural (b) gradient values of all fifteen function networks were summarized using box plots ordered by the mean value. On each box plot, the central mark indicates the mean, and the bottom and top edges of the box indicate the 25th and 75th percentiles, respectively. The whiskers extend to the most extreme data points not considered outliers. The abbreviation of marmoset networks was summarized in Supplementary Table 1. Sample size for each box plot was the number of voxels in each marmoset network and summarized in Supplementary Table 1.

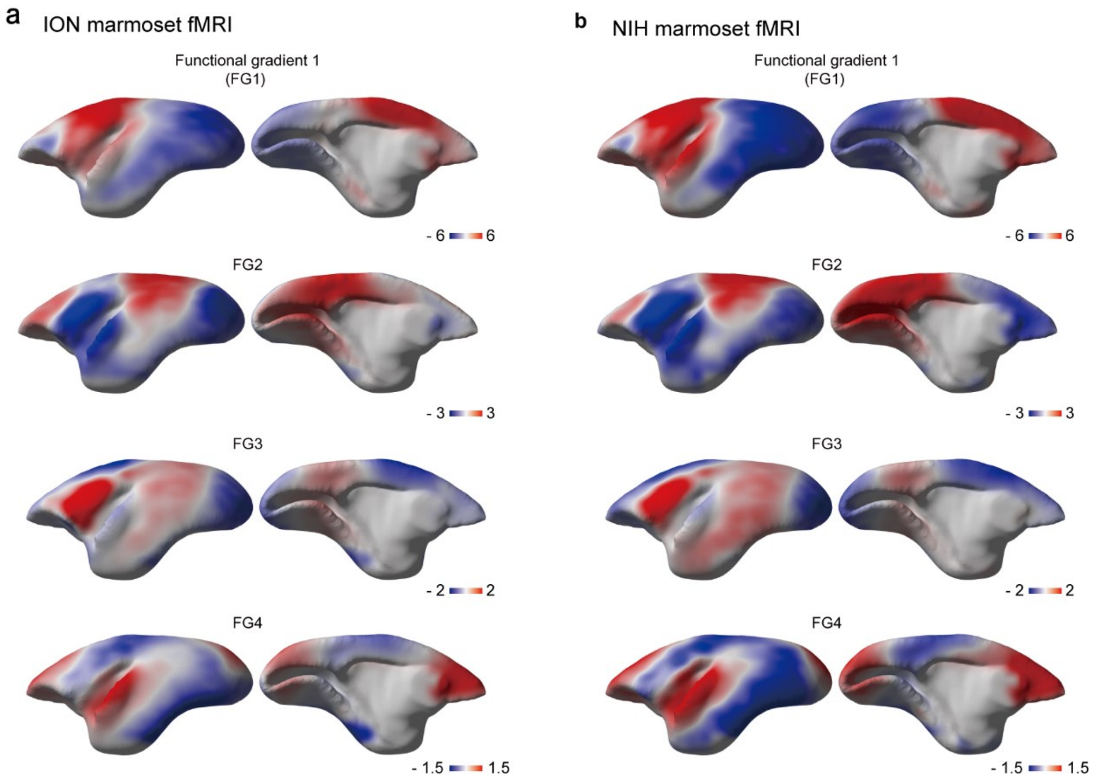

Supplementary Fig. 3 Reproducibility of marmoset functional gradients.
(a-b) First four gradients of marmoset function connectivity from ION (a) and NIH (b) fMRI datasets. FG, functional gradient.
Source data are provided as a Source Data file.

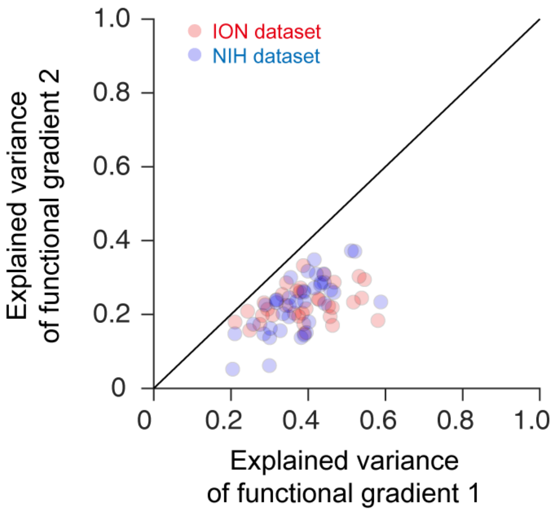

Supplementary Fig. 4 Stable order of functional gradient across individual marmosets.

The explained variance of functional gradient 1 was consistently higher than that of functional gradient 2 across individual marmosets. Each dot represented an individual marmoset. N = 13 marmoset for ION dataset, and N = 26 marmosets for NIH dataset. Source data are provided as a Source Data file.

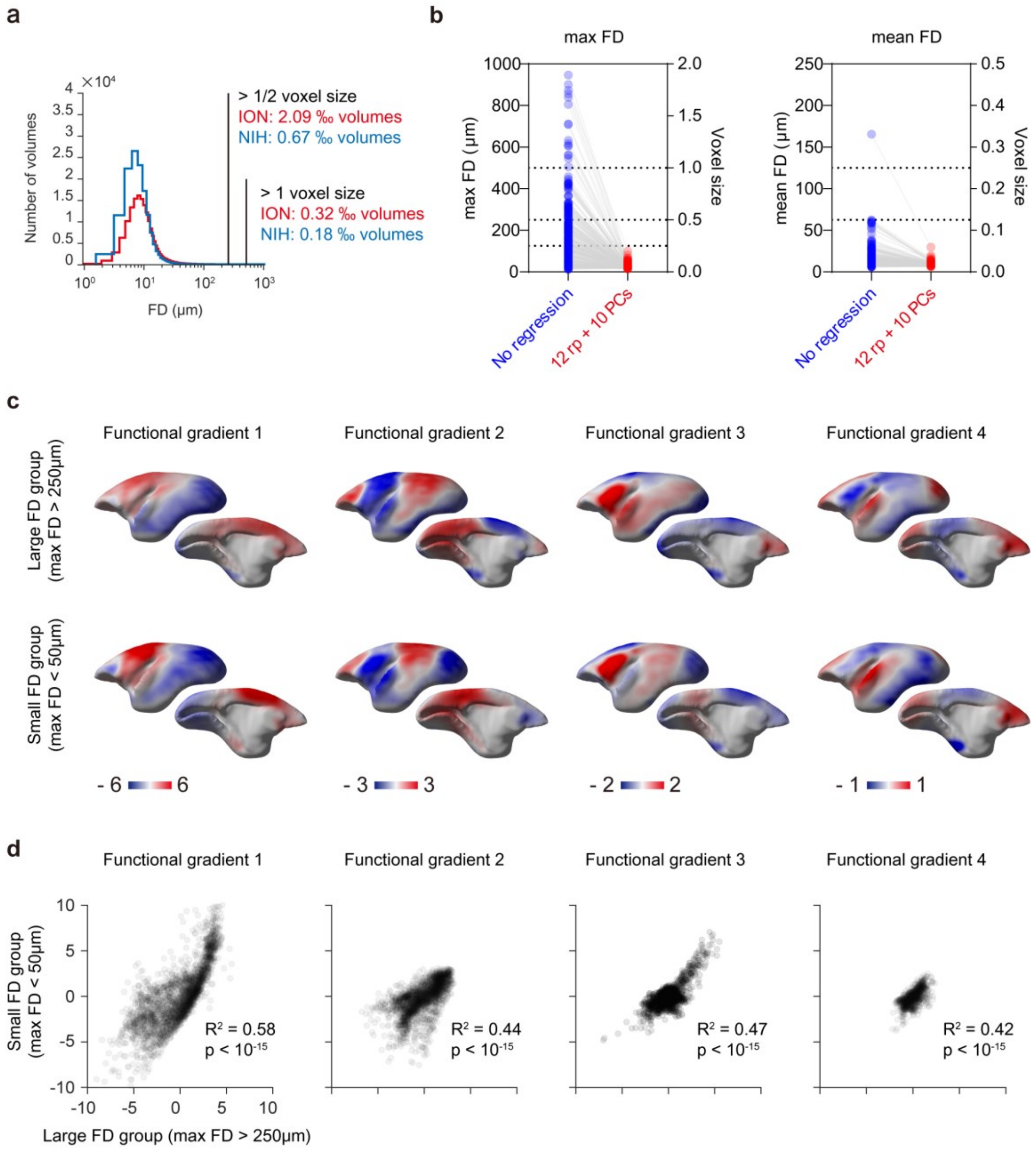

Supplementary Fig. 5 Head motion had minimal impact on gradient results after preprocessing.

(a) Distribution of frame-wise displacement (FD) across marmoset raw EPI volumes.

(b) Effect of marmoset head motion removal after the “12 rp + 10 PCs” regression. Each dot represented an EPI run.

(c-d) Reproducibility (c) and high inter-group similarity (d) of marmoset functional gradients between large (FD>250 μm) and small (FD<50 μm) head motion groups (two-tailed *t*-test). Each dot represented a voxel of marmoset brain.

Source data are provided as a Source Data file.

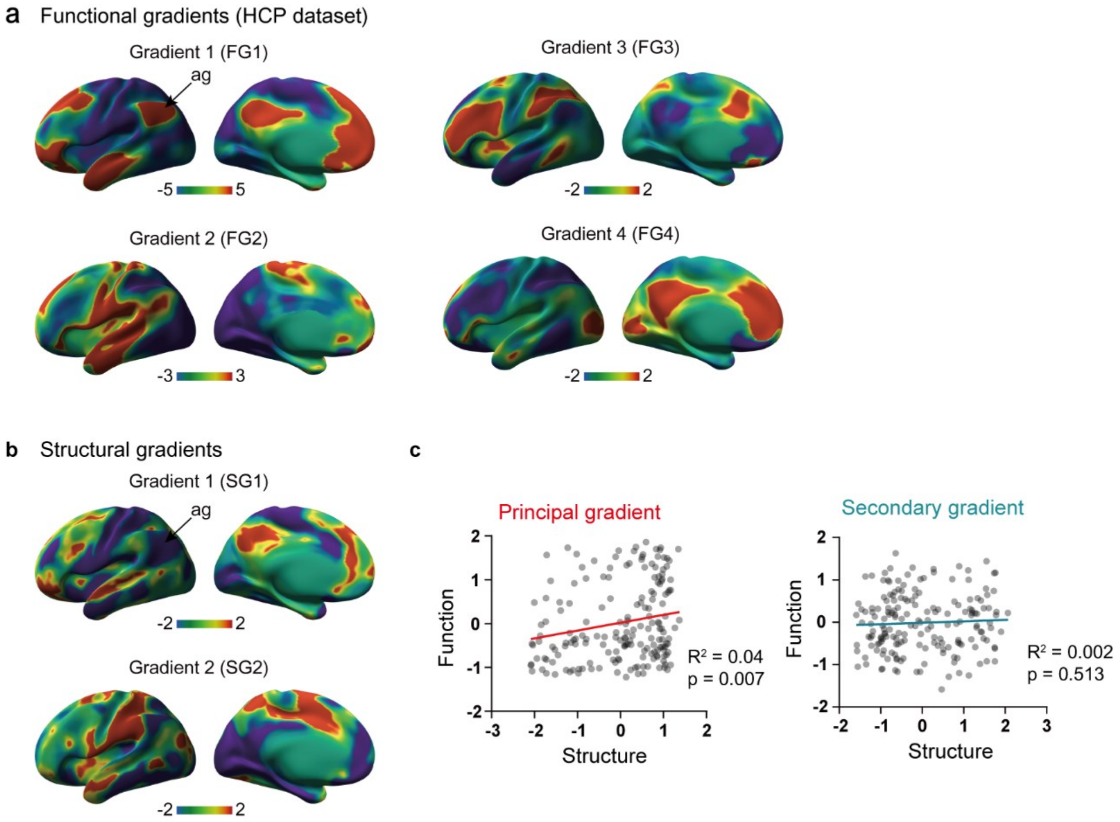

Supplementary Fig. 6 Spatial topography of human functional and structural gradients.

(a) The first four human functional gradients (adults, HCP dataset). FG, functional gradient. ag, angular gyrus.

(b) The first two human structural gradients. The structural connectivity matrix was adopted from Rosen et al. (doi:10.1523/ENEURO.0416-20.2020) SG, structural gradient.

(c) Weak spatial similarity between structural and functional gradients (two-tailed *t*-test). Each dot represents a brain region.

Source data are provided as a Source Data file.

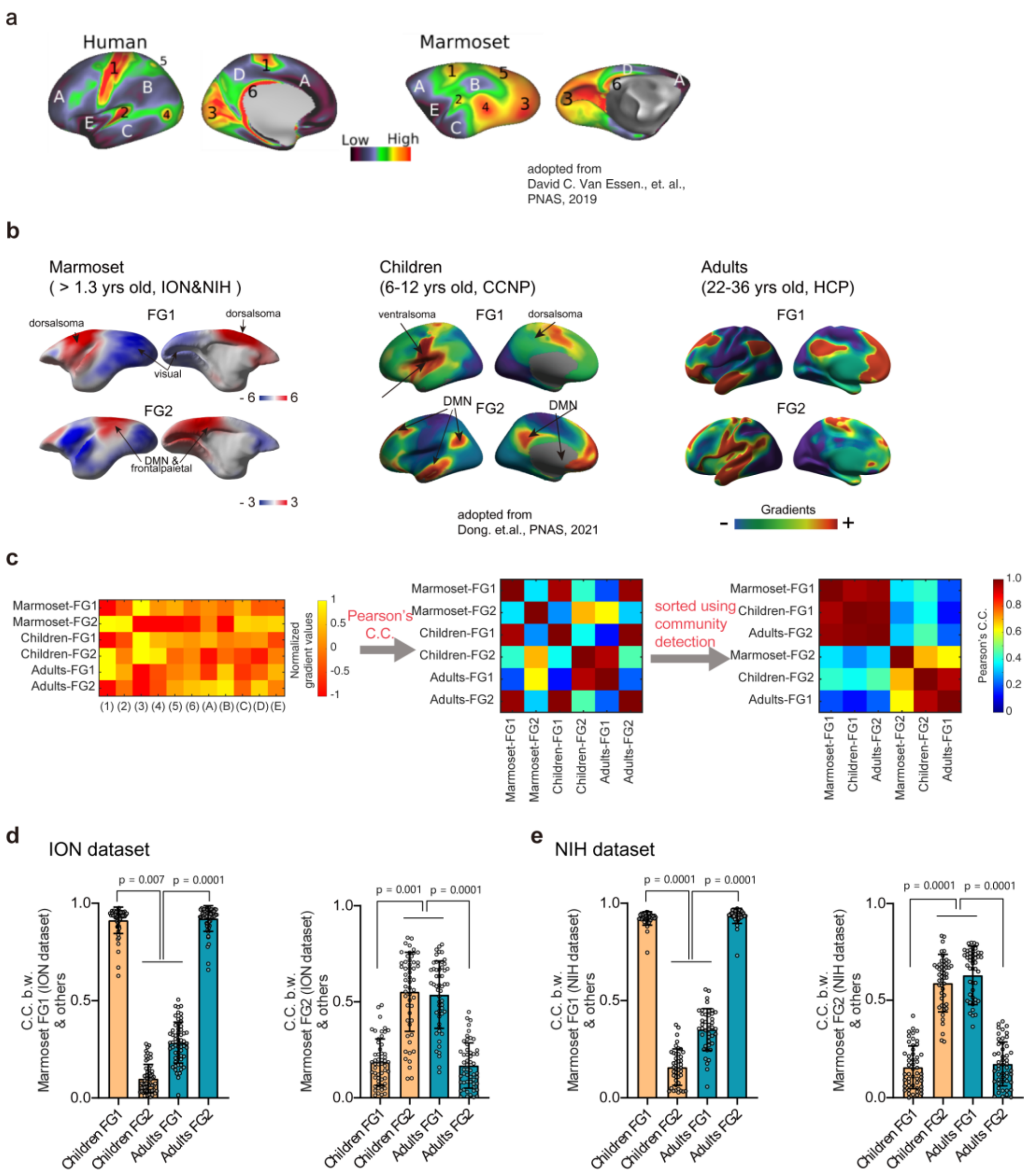

Supplementary Fig. 7 Marmoset functional gradients closely resembled children functional gradients in human.

(a) Eleven cross-species landmark regions for gradient fingerprint analysis. The figure was adapted from Van Essen, et. al., PNAS, 2019. (doi:10.1073/pnas.1902299116)

(b) The first two functional gradients (FG) of marmoset, human children and adults.

(c) Marmoset functional gradient closely resembled that of human children. Based on gradient values from 11 cortical landmark regions, we extracted the cross-species gradient fingerprints (upper) and calculated the Pearson's correlation coefficients (C.C.) between each pair of gradient fingerprints. Using the method of community detection, we clustered 2 communities of cross-species gradients, suggesting the marmoset functional gradients closely resembled the children cortical organization of human.

(d-e) Marmoset FG 1 was more correlated with children FG 1 and adult FG 2 than adult FG 1, and Marmoset FG 2 was more correlated with children FG 2 and adult FG 1 than adult FG 1 in both ION (d, n = 56 sessions) and NIH (e, n = 47 sessions) datasets. Error bar, Mean +/- STD. Two-tailed *t*-test. Source data are provided as a Source Data file.

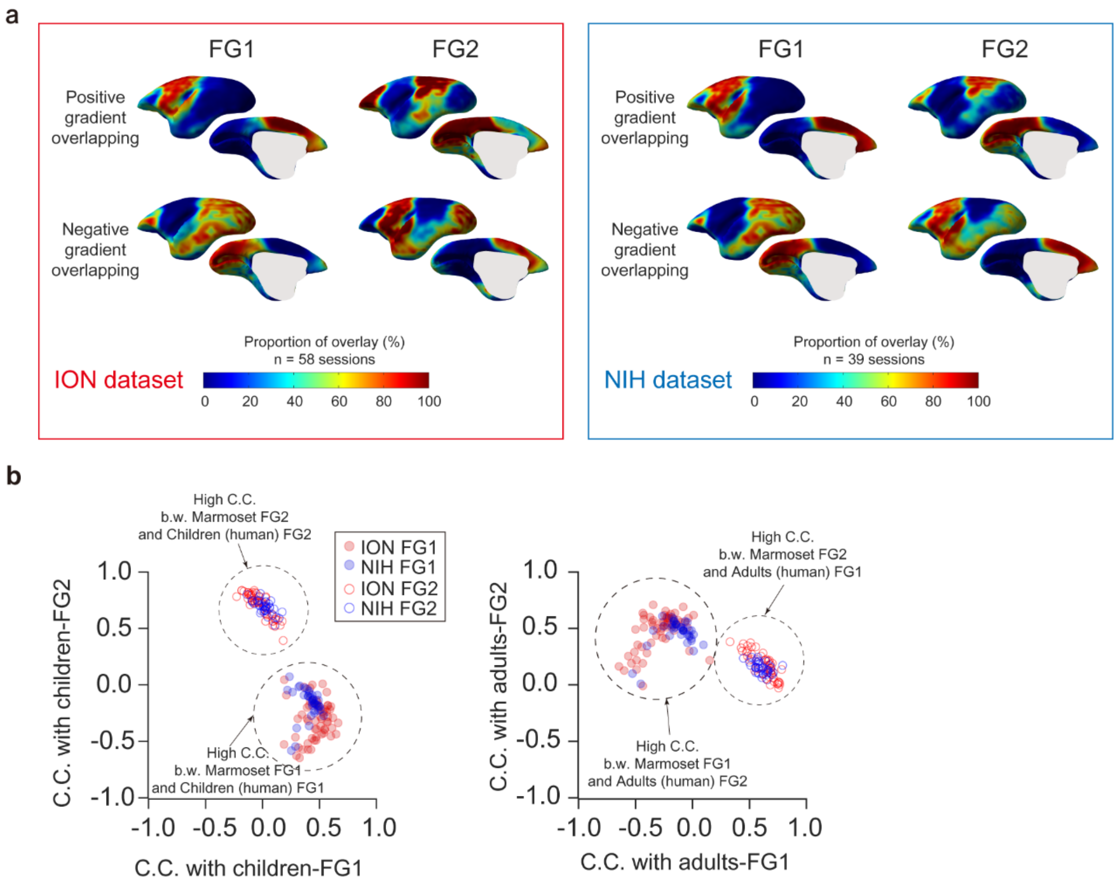

Supplementary Fig. 8 Highly reproducible similarity between averaged human (children and adults) gradients and individual marmoset gradients.
(a) High stability of functional gradient patterns at the individual session level in both ION and NIH dataset. FG, functional gradient.
(b) Highly reproducible similarity between averaged human (children and adults) gradients and individual marmoset gradients. Each dot represented an individual marmoset EPI session. C.C., Pearson's correlation coefficients.
Source data are provided as a Source Data file.

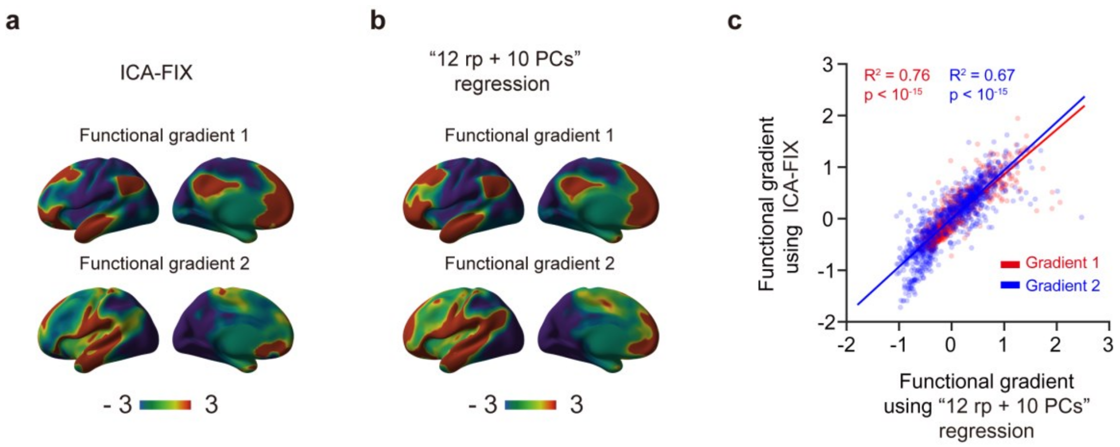

Supplementary Fig. 9 Pre-processing strategies did not significantly influence the results of functional gradients.

(a-b) The human functional gradients with different preprocessing strategies, i.e., ICA-FIX (HCP) and “12 rp + 10 PCs” regression (used in marmoset preprocessing strategy).

(c) Significant correspondence between the functional gradients with the two different preprocessing strategies in human HCP data (two-tailed *t*-test).

Source data are provided as a Source Data file.

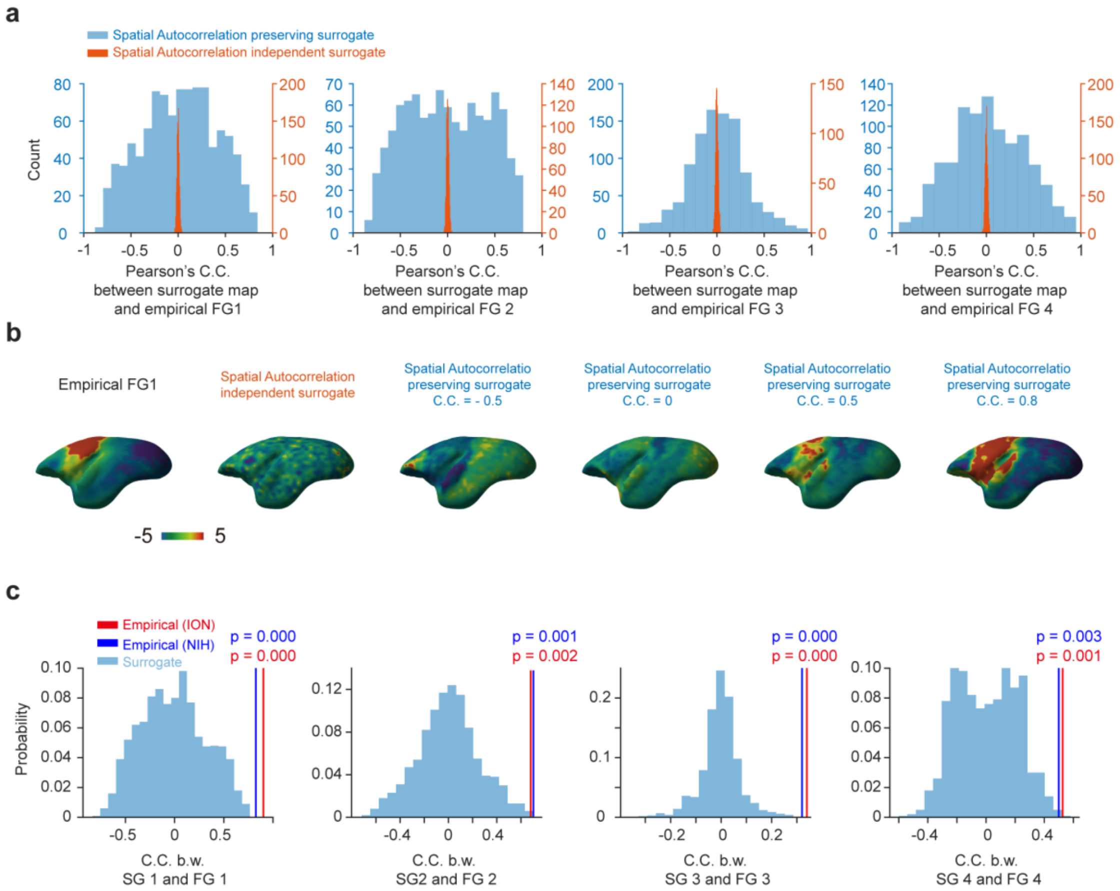

Supplementary Fig. 10 Spatial autocorrelation (SA) preserving surrogate maps provided a more conservative and meaningful measure of statistical significance for the similarity of structural-functional gradients.

(a) Histogram of spatial correlation between surrogate maps and empirical one. SA preserving surrogate maps showed wider distribution of correlation with the empirical one, compared to the SA independent surrogate maps.

(b) The empirical functional gradient 1 map and example surrogate maps with matched independent and preserving spatial autocorrelation.

(c) Null distributions of Pearson's correlations between structural gradients and functional surrogate gradients (sky blue, 1000 shuffled SA-preserving). Red and blue lines represented the Pearson's correlation (two-tailed *t*-test) between empirical structural and functional gradients.

Source data are provided as a Source Data file.

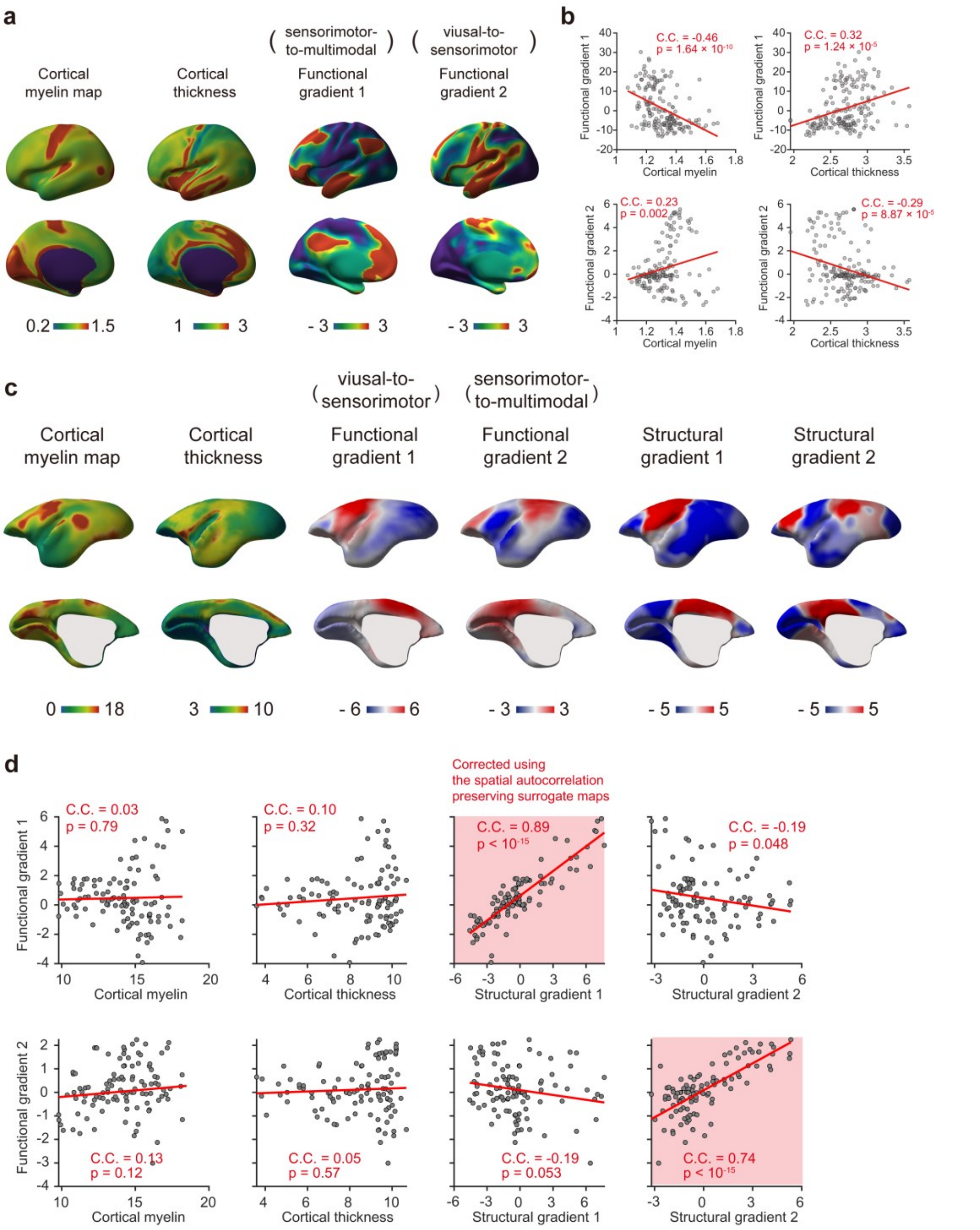

Supplementary Fig. 11 Cortical thickness and myelin map are significantly correlated with functional gradients in human but not in marmoset.

(a) Spatial profiles of human cortical myelin, thickness and first 2 functional gradient maps. (b) Significant correlation between cortical myelin (or thickness) map and human functional gradients (two-tailed *t*-test). Each dot represented a brain region. Red line was the best linear fit. (c) Spatial profiles of marmoset cortical myelin, thickness, first 2 functional and structural and gradient maps. (d) Functional gradients in marmosets exhibited significant correlation with structural gradients (two-tailed *t*-test), but not with the cortical thickness and myelin maps. Each dot represented a brain region. Red line was the best linear fit.

Source data are provided as a Source Data file.

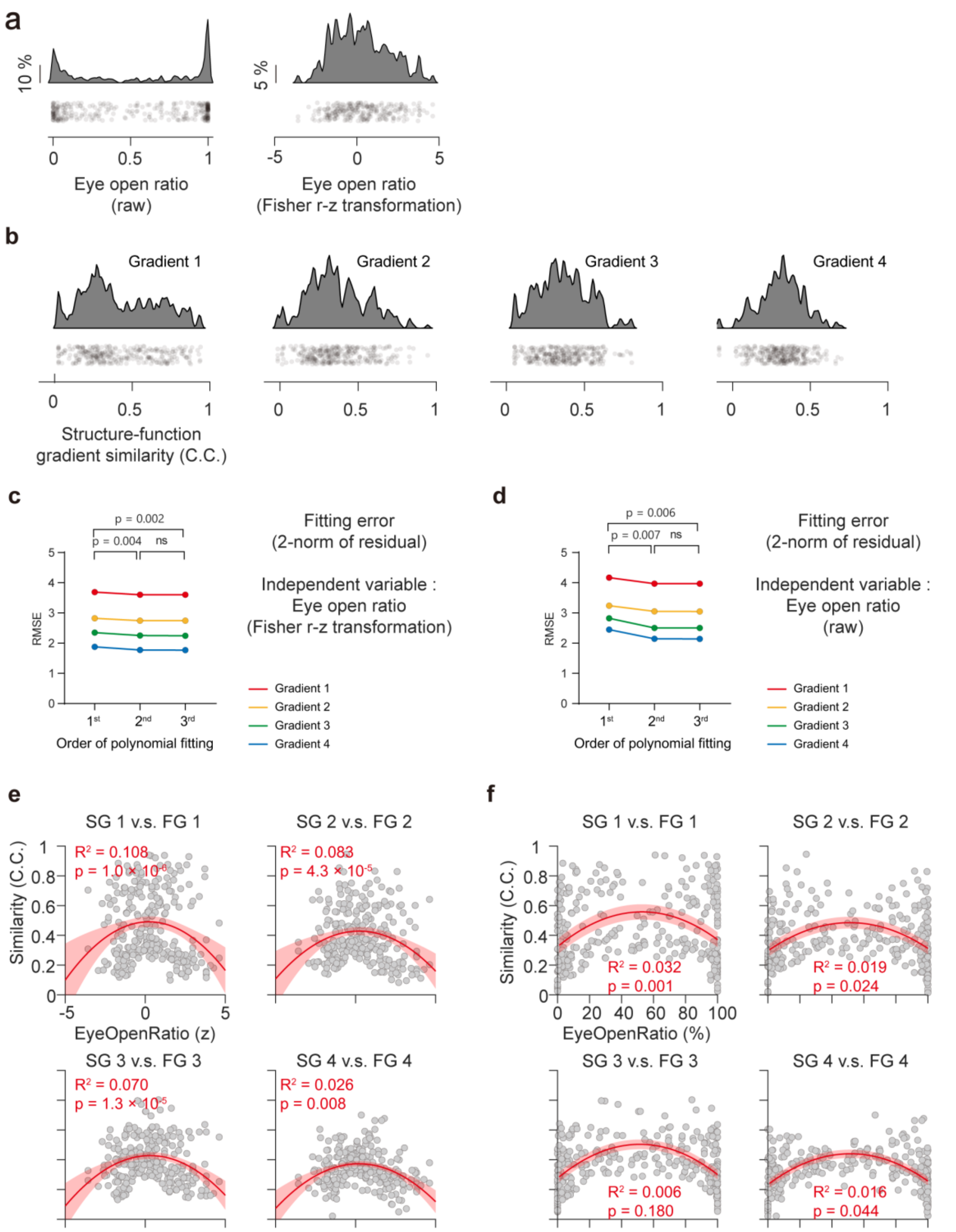

Supplementary Fig. 12 Consistent inverted U-shape relationship between Fisher transformed eye open ratio and structure-function gradient similarity.

(a) Distribution of marmoset behavior arousal index, i.e., eye open ratio. The raw eye open ratio showed a “semi- Beta” distribution, while the Fisher transformed eye open ratio showed a gamma-like distribution. Notably, scans with fully eye open or closed were excluded for the Fisher r-z transformation. Each dot represented a scan. (b) Gamma-like distributions of first four structure-function gradient similarity. Each dot represented a scan.

(c-d) The fitting error among 1st-, 2nd- and 3rd- order polynomial fitting between Fisher transformed (c) or raw (d) eye open ratio and structure-function gradient similarity. Two-tailed *t*-test. (e-f) 2nd- order polynomial fitting between Fisher transformed (e) or raw (f) eye open ratio and structure-function gradient similarity (two-tailed *t*-test). Each dot represented a scan. Red line indicated the fitting curve. Red shade represented the 95% prediction interval.

Source data are provided as a Source Data file.

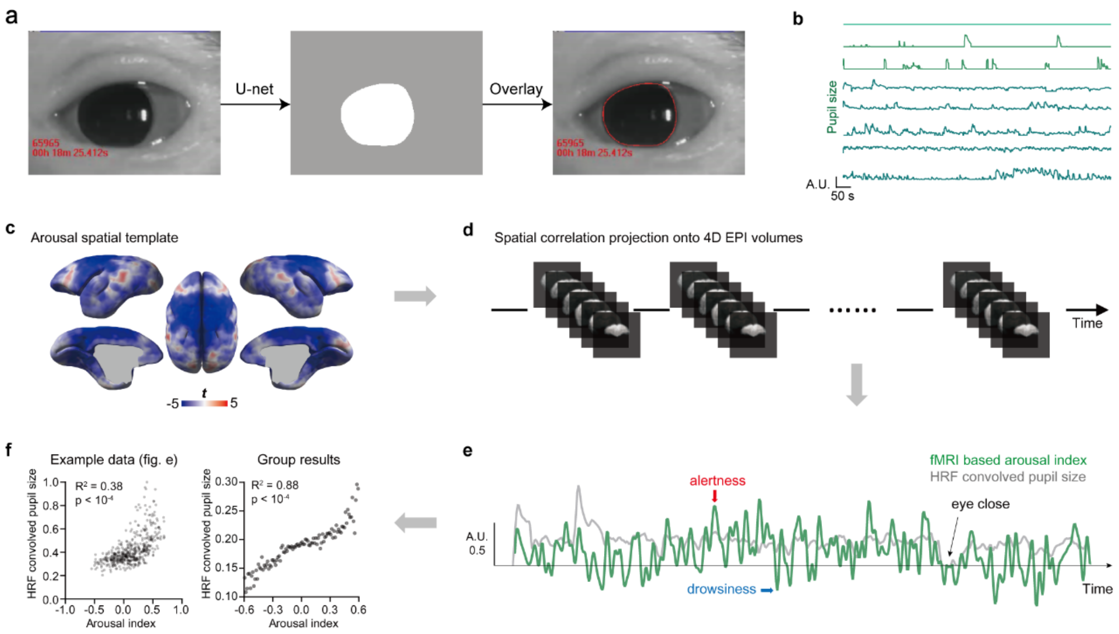

Supplementary Fig. 13 fMRI based arousal index in marmoset.

(a) Pupil size extraction utilizing the U-net segmentation.

(b) Examples of marmoset pupil size variation during MRI scanning.

(c) Spatial pattern of correlation between the time course of HRF convolved pupil size and the resting-state fMRI signal fluctuations of each voxel.

(d-f) Pipeline for estimating the fMRI based arousal index. The fMRI based arousal index was defined as spatial correlation between the arousal template map (c) and each successive fMRI frame (d). The fMRI based arousal index exhibited high performance on individual scan (e and left panel of f, $p = 3.6 \times 10^{-8}$, two-tailed *t*-test) and group results (f right panel; $p = 1.2 \times 10^{-10}$, two-tailed *t*-test).

Source data are provided as a Source Data file.

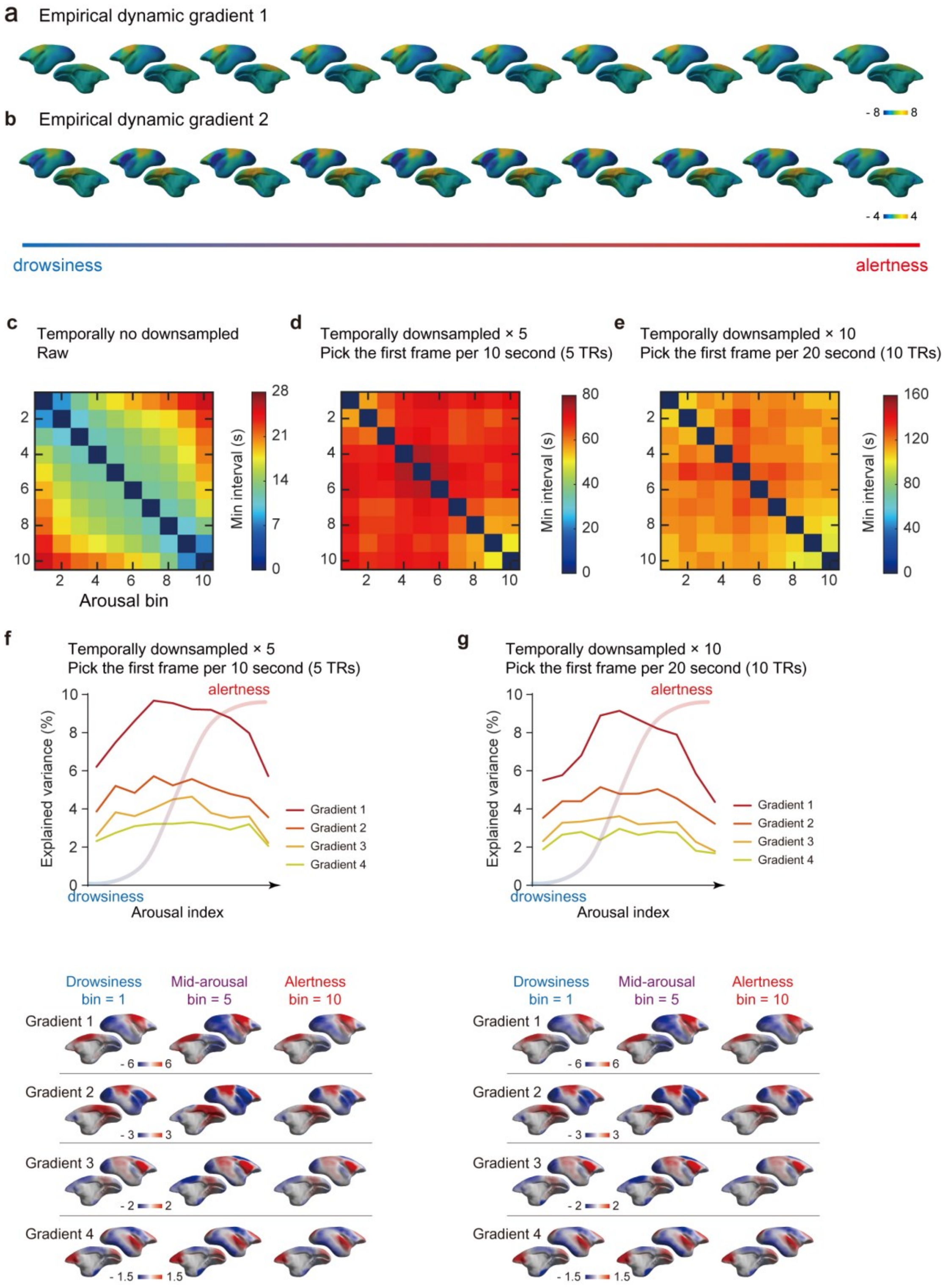

Supplementary Fig. 14 Arousal relevant functional gradient dynamics.

(a-b) The arousal relevant gradient values of first two gradients. Gradient values varied with arousal fluctuations.

(c) Averaged minimum temporal intervals among different arousal bins across EPI runs. Large temporal gaps (25.5 s) was shown between extremely low (bin=1) and high (bin=10) arousal levels.

(d-e) Apparent increases of averaged minimum temporal intervals among different arousal bins with 5 times (d) or 10 times (e) down-sampled time points.
(f-g) Consistent inverted U-shape relationship between gradient explained variances and 5 times (f) or 10 times (g) down-sampled time points. (Top) inverted U-shape relationship between arousal level and explained variance across gradients. (Bottom) highly stable gradient profiles across arousal levels.
Source data are provided as a Source Data file.

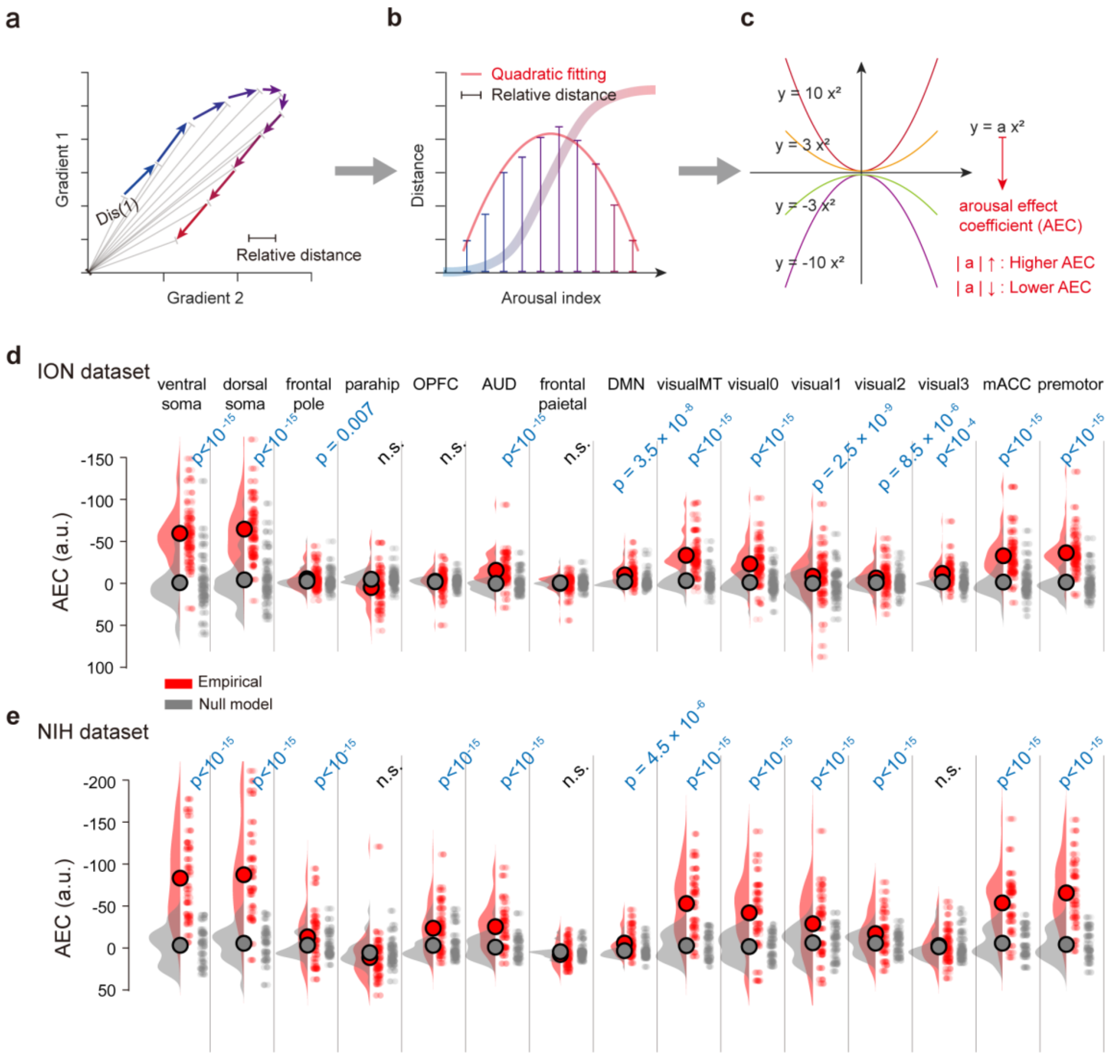

Supplementary Fig. 15 Quantitative evaluation of functional gradient “flood and ebb” dynamics with arousal fluctuations.

(a-c) Definition of arousal effect coefficient (AEC). For each marmoset brain region, we extracted the relative distances (to the origin of gradient space) along with arousal fluctuations (a) and made a 2nd-order polynomial fitting to the relative distances (b). We quantitatively defined the polynomial coefficient ***a*** as arousal effects coefficient (AEC) of functional gradient dynamics (c). Higher AEC indicates sharper U-shape (or inverted U-shape) relationship, corresponding to a higher arousal contribution. (d) Significant shift of arousal relevant gradient flow in most regions compared to the null model in the ION dataset (two-tailed *t*-test). The arousal index was shuffled by scan, i.e., replacing the arousal index of scan A by that of scan B, and then same analysis was applied to generate the null model control. Each dot represented an individual EPI scan. (e) As in (d) but for the NIH dataset. n.s., no significance.

Source data are provided as a Source Data file.

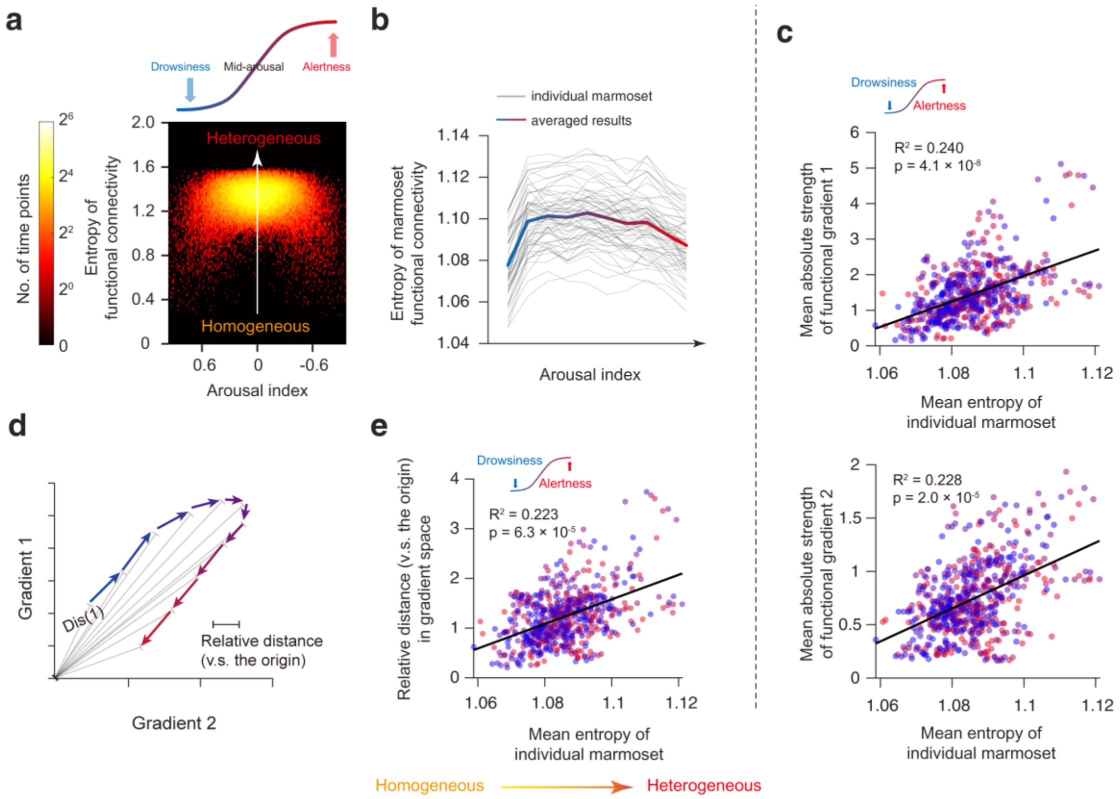

Supplementary Fig. 16 Gradient dynamics is related to the heterogeneity of functional connectivity with arousal fluctuations.

(a) Inverted U-shape relationship between arousal index and the entropy of dynamic functional connectivity (FC). Higher entropy indicated more heterogeneous functional connectivity.

(b) Stability of the inverted U-shape relationship between arousal index and the entropy of dynamic FC across individual marmosets.

(c) Significant correlation (two-tailed *t*-test) between the mean entropy of dynamic FC and the mean whole brain absolute strength of functional gradient 1 (upper panel) and 2 (lower panel) of individual marmosets across arousal levels (10 bins).

(d) Illustration of the relative distance of a particular brain region (v.s. the origin) in gradient space.

(e) Entropy of dynamic FC contributed to the “ebb and flow” effect in gradient space. Significant correlation between the mean entropy of dynamic FC and the mean whole brain relative distance in the gradient space (v.s. the origin) across individual marmosets (two-tailed *t*-test). Each dot represent an individual marmoset across arousal levels (10 bins).

Source data are provided as a Source Data file.

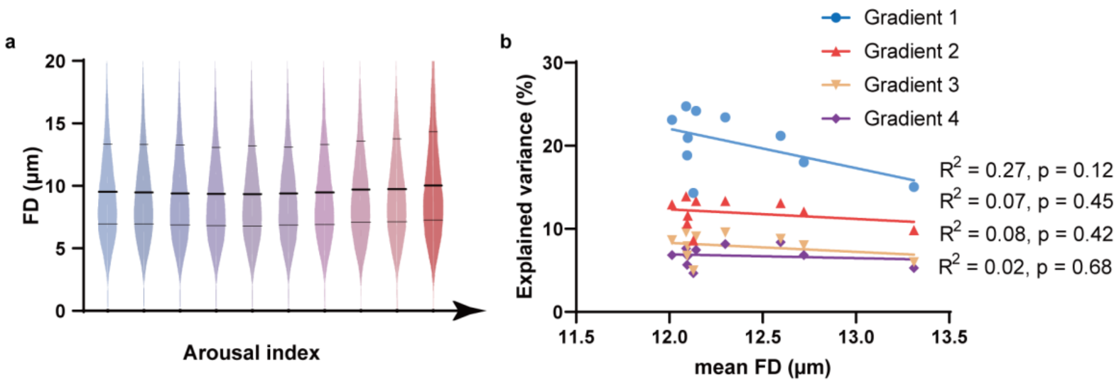

Supplementary Fig. 17 No significant correlation between head motion and gradient dynamics

(a) Frame-wise displacements (FD) across arousal levels, yielding slight increases of frame-wise displacement from drowsiness to alertness. Data were presented by the violin plots (n = 31835 +/- 5 time points), in which the bold lines represented the median value and thin lines represented the 25% and 75% quartiles, respectively.

(b) No significant correlation between mean frame-wise displacement and arousal relevant explained variances across arousal levels (two-tailed *t*-test). Each dot represented an arousal bin.

Source data are provided as a Source Data file.

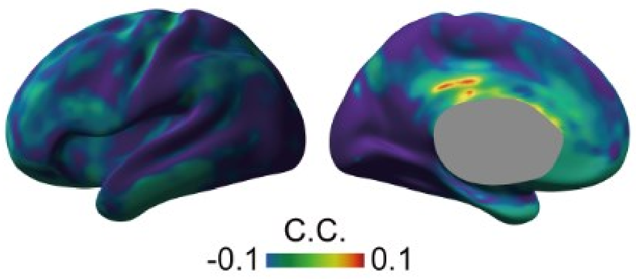

Supplementary Fig. 18 Human arousal spatial template.

Negative correlation between EEG-based arousal index and fMRI signal. The correlation map, i.e., human arousal spatial template, was modified from Falahpour et al. (doi:10.1016/j.neuroimage.2018.03.012) C.C., correlation coefficients.

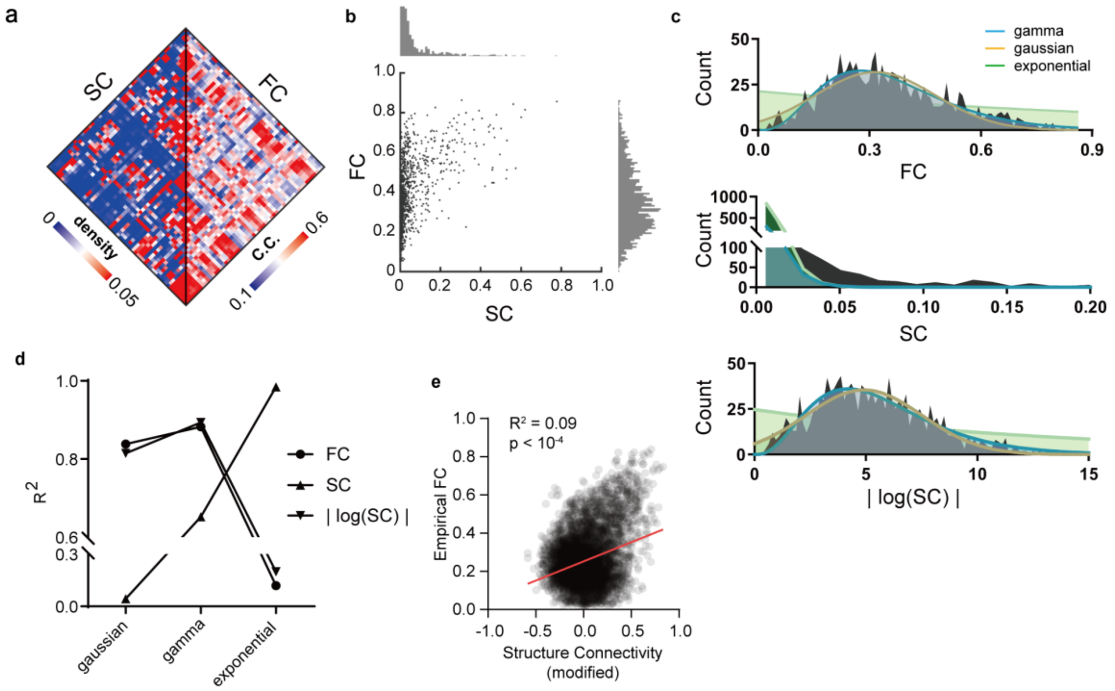

Supplementary Fig. 19 Optimized structure connectivity preferably captured the function connectivity, compared with the raw structure connectivity in marmoset.

(a) Side-by-side comparison of structure connectivity (SC) and function connectivity (FC). The structure connectivity was drawn from a recently published marmoset retrograde tracing matrix (http://marmoset.brainarchitecture.org/).

(b) Scatter plots between FC and SC. The right and upper inserts showed the distribution of FC and SC, respectively.

(c) FC exhibited gamma-like distribution, whereas the SC exhibited exponential-like distribution. The distribution of |log(SC)| was similar to the distribution of FC.

(d) The goodness of fitting for FC, SC and |log(SC)| showed an independent identically distributed (i.i.d.) structure. Notably, the structure information used in our marmoset GLM model was the optimized structure connectivity only, i.e., |log(SC)|.

(e) Significant correlation between structure connectivity and empirical function connectivity (two-tailed *t*-test). The modified structure connectivity was derived from the region-wise correlation of |log(SC)|.

Source data are provided as a Source Data file.

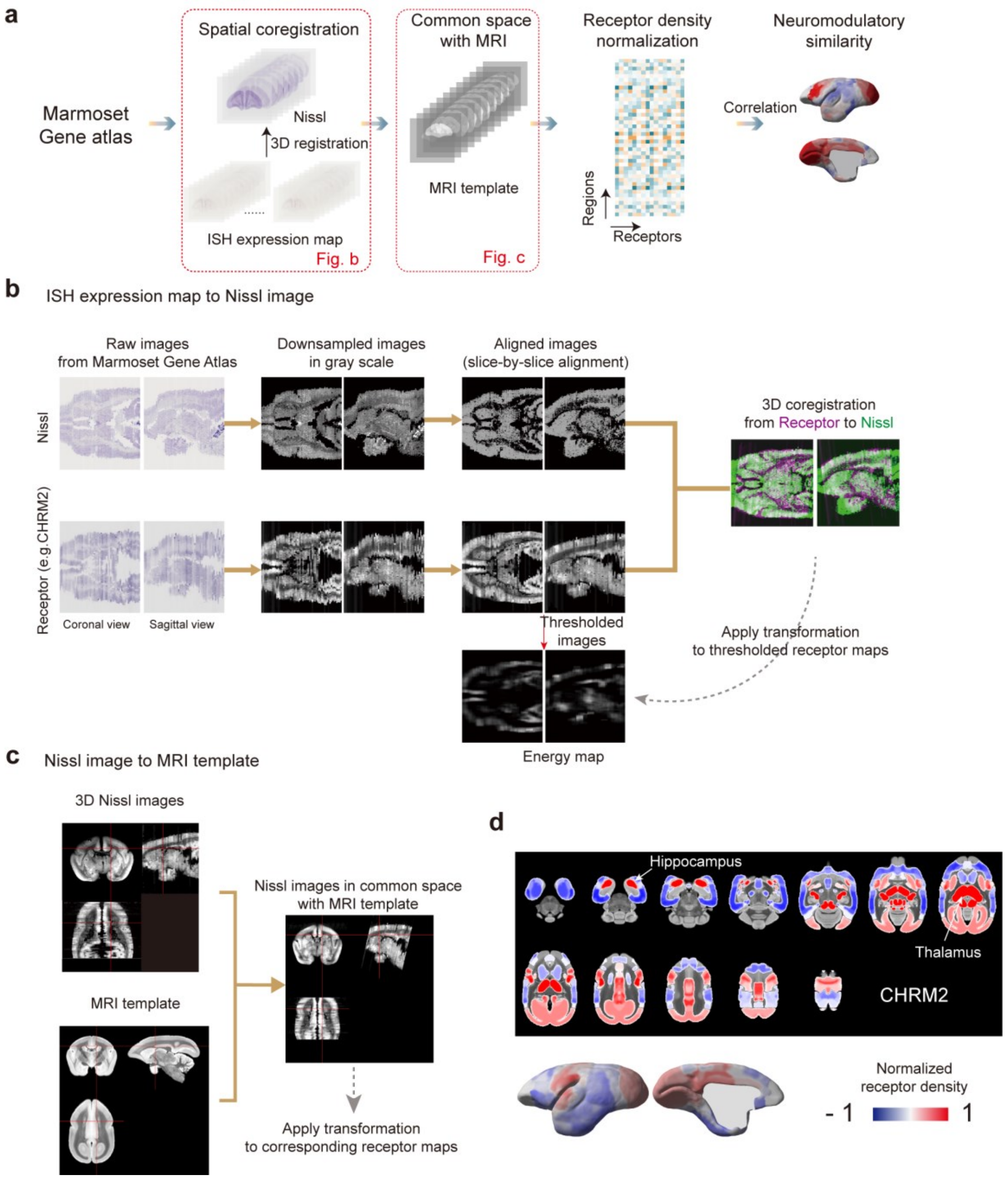

Supplementary Fig. 20 Registration of marmoset ISH images to MRI space. (a) Computational strategy of marmoset ISH registration and generation of neuromodulatory receptor similarity. The neuromodulatory receptor gene expression data were obtained from the marmoset gene atlas database (https://gene-atlas.brainminds.riken.jp/) . Briefly, we downloaded neuromodulatory receptor related gene expression maps and the corresponding Nissl stained coronal images. Next, we registered the expression maps to the Nissl stained images, yielding a 3D spatial alignment between anatomical and gene expression images (details in Fig. b). The Nissl stained images were registered to the study-specific MRI template (details in Fig. c), and this transformation was applied to the gene expression maps to bring them to the MRI space. Gene expression data were then parcellated into 116 cortical regions of interest, based on the Riken

Brain/MINDS cortical parcellation. Finally, the resulting neuromodulatory receptor similarity was calculated using the correlation of normalized gene expression levels across each pair of regions.
(b) Registration from receptor gene expression maps to Nissl images. Firstly, these ISH images were down-sampled (5 times) in gray scale to reduce the computation load. Then, these Nissl and receptor expression images were iteratively aligned to a weighted average of its neighbors, respectively. The receptor expression images were de-noised using the standard medial filter and further threshold by the median values of the ISH images, termed as the "energy map" (bottom image in b). Finally, the 3D receptor expression maps were registered to the Nissl images using rigid-body transformation, and the transformation matrix was applied to the receptor energy maps. Further adjustment of registration was made slice-by-slice using the method of large deformation diffeomorphic metric mapping (LDDMM).
(c) Cross-modal registration from Nissl images to MRI template. The 3D Nissl images were nonlinearly transformed to the MRI template using the "oldnormalize" of the SPM12. The affine transformation was then applied to the receptor expression energy map.
(d) Example of registered and normalized CHRM3 receptor expression energy map. The expression energy was overlaid on the MRI template in coronal slice view (upper panel) and 3D surface view (lower panel).

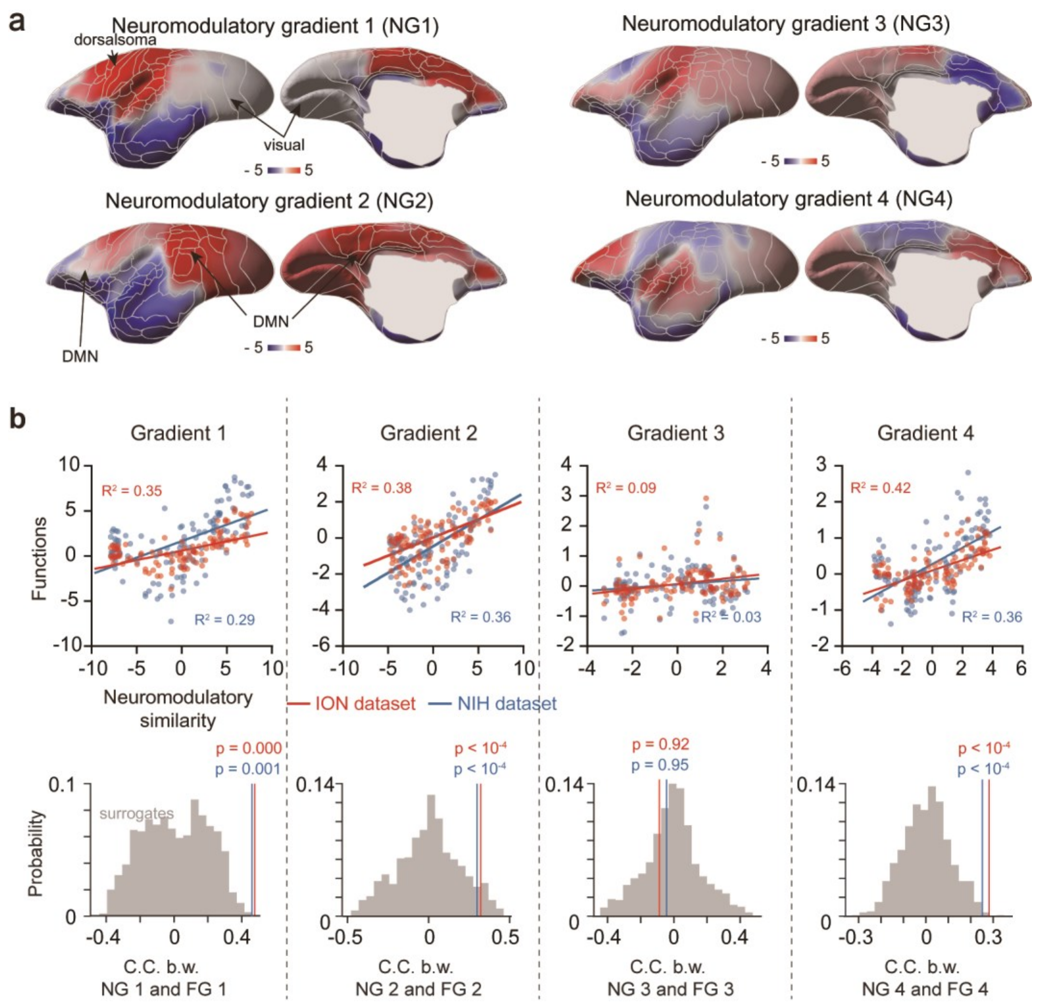

Supplementary Fig. 21 Gradients of neuromodulatory similarity in marmoset.

(a) First four gradients of marmoset neuromodulatory similarity based on marmoset gene atlas. Areal borders were based on the Riken Brain/MINDS cortical parcellation. NG, neuromodulatory gradient.

(b) Topographical similarity between neuromodulatory and functional gradients. High similarity between neuromodulatory and empirical functional gradients (upper panel). Significant difference between the empirical similarity of neuromodulatory-functional gradients and null distributions (lower panel), in which null distributions was derived from the Pearson's correlation coefficients (gray shades) between neuromodulatory gradients and the spatial autocorrelation preserving surrogate maps of functional gradients. Each dot represented a brain region. Red and blue lines represented the Pearson's correlation (two-tailed *t*-test) between empirical neuromodulatory and functional gradients.

Source data are provided as a Source Data file.

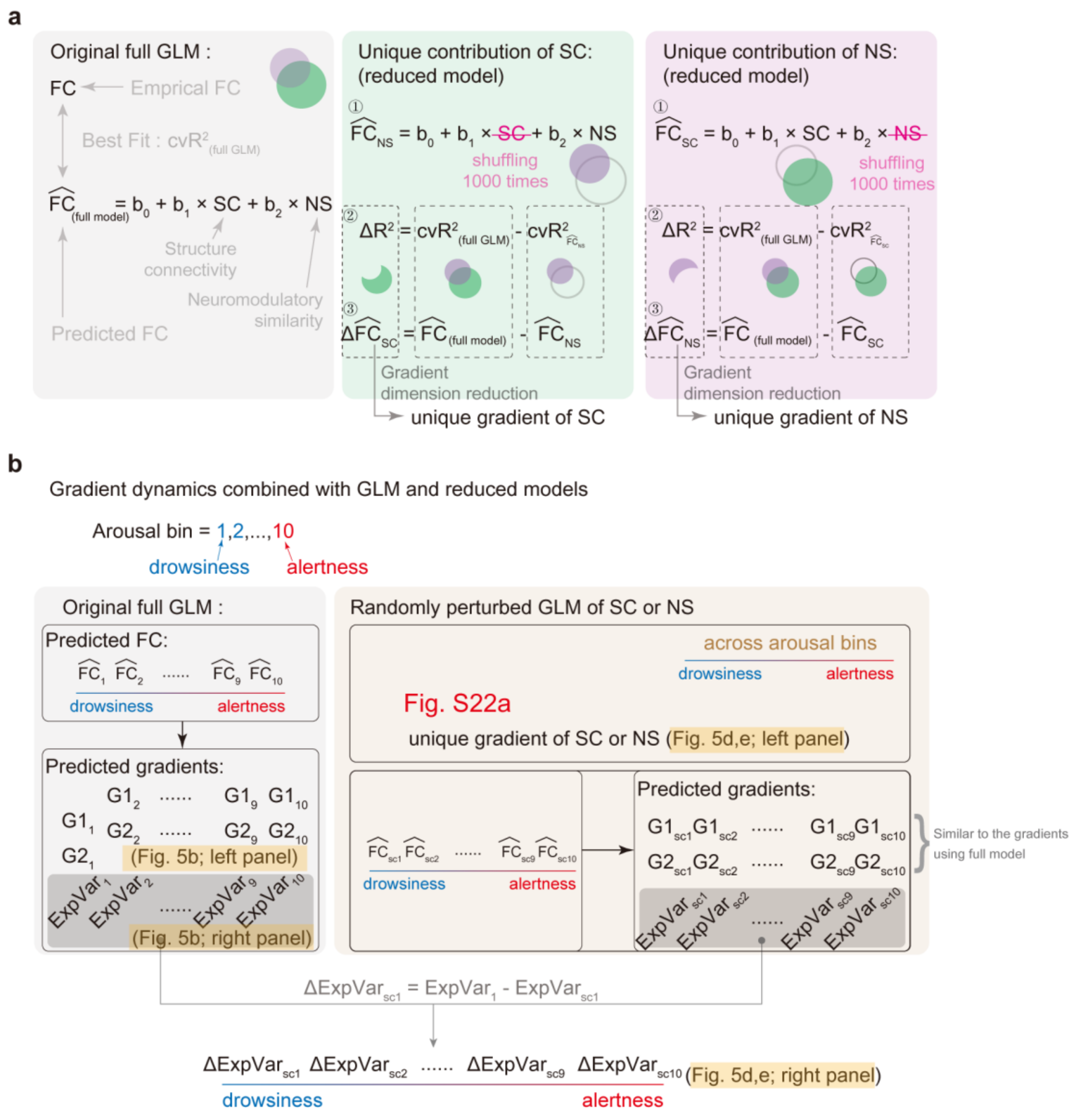

Supplementary Fig. 22 Computational pipeline of gradient (dynamics) using general linear model (GLM) and reduced model

(a) In the reduced model, the variable of interest was randomly shuffled 1000 times, and the resulting loss of explained variance (compared to the full model) was the unique contribution of this particular variable in the reduced model. SC, structural connectivity; NS, neuromodulatory similarity.

(b) Firstly, for the full general linear model (GLM), we calculated the predicted FC across arousal bins, and obtained the first two gradients and corresponding ExpVar (left panel). Because of the highly spatial similarities of the gradients across arousal bins, we only presented the gradient profiles in mid-arousal level (bin=5). Then, for the unique contribution of functional gradients of SC or NS, we conducted similar analyses compared to full GLM. The unique gradient profiles was derived from the difference ($\Delta\widehat{FC}$) between the full GLM model ($\widehat{FC}$) and the reduced model ($\widehat{FC_{SC}}$) (right upper panel). Finally, the $\widehat{FC_{SC_{1,2,\ldots,10}}}$ were further decomposed into a set of gradients and corresponding ExpVars. The unique contribution of explained variance (ΔExpVar) was the ExpVar difference between full GLM and reduced model (right lower panel).

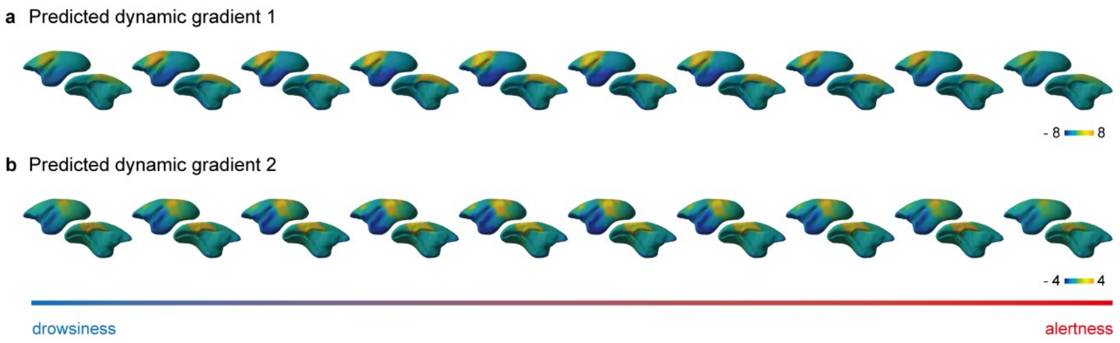

Supplementary Fig. 23 High accuracy of dynamic gradient modelling.

(a-b) Arousal relevant topographies of predicted functional gradient dynamics. Gradient values displayed the tendency of rising up at drowsiness state and declining to alertness state, similar to the empirical results.

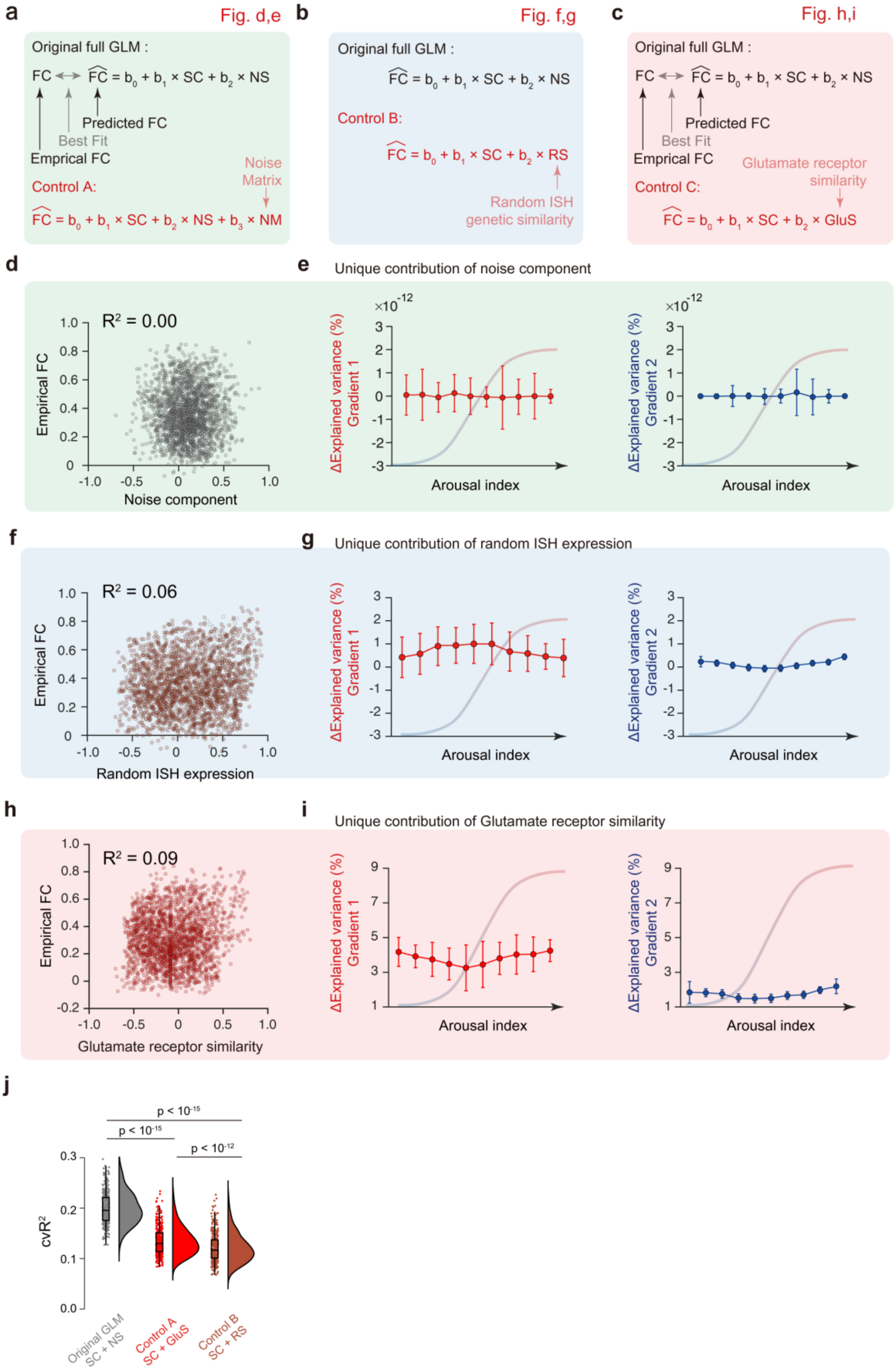

a
Fig. d,e
Original full GLM :
FC ↔ $\widehat{FC} = b_0 + b_1 \times SC + b_2 \times NS$
Predicted FC
Best Fit
Emprical FC
Noise Matrix
Control A:
$\widehat{FC} = b_0 + b_1 \times SC + b_2 \times NS + b_3 \times NM$
b
Fig. f,g
Original full GLM :
$\widehat{FC} = b_0 + b_1 \times SC + b_2 \times NS$
Control B:
$\widehat{FC} = b_0 + b_1 \times SC + b_2 \times RS$
Random ISH genetic similarity
c
Fig. h,i
Original full GLM :
FC ↔ $\widehat{FC} = b_0 + b_1 \times SC + b_2 \times NS$
Predicted FC
Best Fit
Emprical FC
Glutamate receptor similarity
Control C:
$\widehat{FC} = b_0 + b_1 \times SC + b_2 \times GluS$
d
$R^2 = 0.00$
Empirical FC
Noise component
e
Unique contribution of noise component
$\times 10^{-12}$
ΔExplained variance (%) Gradient 1
ΔExplained variance (%) Gradient 2
Arousal index
f
$R^2 = 0.06$
Empirical FC
Random ISH expression
g
Unique contribution of random ISH expression
ΔExplained variance (%) Gradient 1
ΔExplained variance (%) Gradient 2
Arousal index
h
$R^2 = 0.09$
Empirical FC
Glutamate receptor similarity
i
Unique contribution of Glutamate receptor similarity
ΔExplained variance (%) Gradient 1
ΔExplained variance (%) Gradient 2
Arousal index
j
$p < 10^{-15}$
$p < 10^{-15}$
$p < 10^{-12}$
cvR^2
Original GLM SC + NS
Control A SC + GluS
Control B SC + RS

Supplementary Fig. 24 Control analysis of the unique U-shape contribution from the neuromodulatory similarity.

(a-c) The control analysis was conducted by (a) adding a noise matrix (NM) as another variable, (b) replacing the NS with random ISH expression similarity (RS) and (c) replacing the NS with glutamate receptor ISH expression similarity (GluS). SC, structural connectivity; NS, neuromodulatory similarity.

(d) Example of a random noise component which did not have a tight link with the empirical functional connectivity.

(e) No significant arousal relevant unique contribution from the noise component. Across arousal levels, the unique contribution was not significantly different from zero (one-sample t-test, two tails). Sample size for each bar plot was 31835 time points (EPI volumes). Error bar, Mean +/- SEM.

(f) Example of the random ISH genetic similarity which showed a significant correlation with empirical functional connectivity.

(g) Inverted U-shape relationship of arousal relevant unique contribution from the random ISH genetic gradient 1, with no arousal relevance on the gradient 2. Sample size for each bar plot was 31835 time points (EPI volumes). Error bar, Mean +/- SEM.

(h) Glutamate receptor similarity showed a significant correlation with the empirical functional connectivity.

(i) U-shape relationship of arousal relevant unique contribution from the random ISH genetic gradient 1 & 2. Sample size for each bar plot was 31835 time points (EPI volumes). Error bar, Mean +/- SEM.

(j) Significant difference (pair-wise t-test) of cross-validated explained variance (cvR^2) from the general linear model (GLM) across the original, control B and control C groups (two-tailed paired *t*-test). Each dot represented an individual EPI runs (n = 709 runs). On each box plot, the central mark indicates the mean, and the bottom and top edges of the box indicate the 25th and 75th percentiles, respectively. The whiskers extend to the most extreme data points not considered outliers.

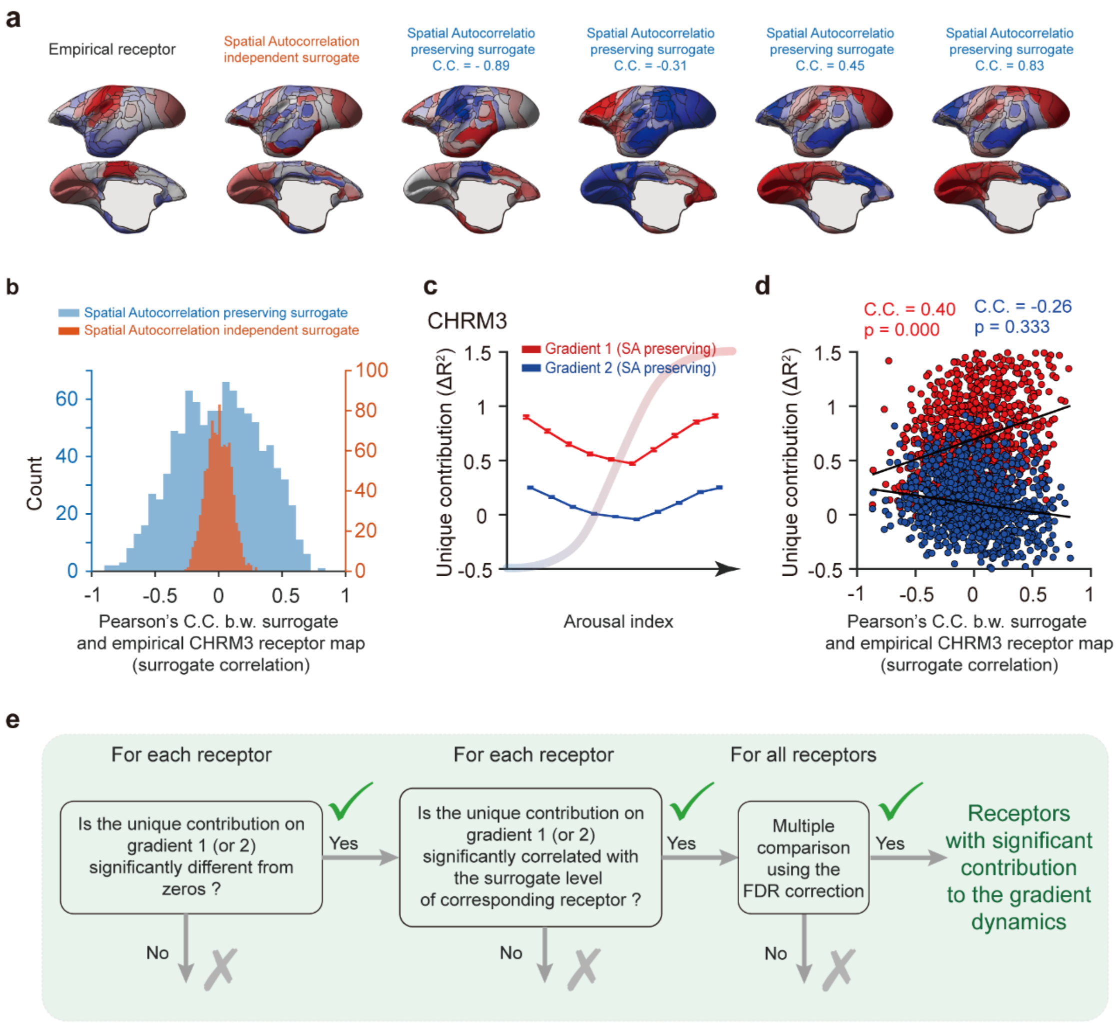

Supplementary Fig. 25 Criterion for identifying the receptors with significant contribution to the arousal relevant gradient dynamics.

Because the neuromodulatory receptors are spatially auto-correlated, we adopted a procedure from previous studies[1-3] (similar to Fig.3C in Demirtas et al., 2019 [3]) to overcome this issue. Thus, we generated surrogate maps that randomly vary in their particular topographies (n = 1000 times shuffling) but preserve the general spatial autocorrelation (SA) structure of corresponding receptors (Fig. a). As expected, the SA preserving surrogate maps showed a much wider distribution of correlation with the empirical one, compared to the SA independent surrogate maps (Fig. b). We calculated the Pearson's C.C. (right tail) between the surrogate similarity (to the empirical one, n = 1000) and the unique contribution on functional gradients of a particular receptor (Fig. c-d, like Fig.3E in Demirtas et al., 2019 [3]). (Errorbar, Mean +/- SEM, n=1000 times shuffling, right-tailed *t*-test) The rationale is: if the correlation is not significant, it means random receptor maps could contribute similar arousal modulation, i.e., the empirical receptor does not specifically contribute to arousal dynamics. Alternatively, if the correlation is significant, it means larger spatial map shuffling causes larger loss of the unique contribution for the corresponding receptor, i.e., the empirical receptor does contribute to arousal dynamics. Finally, we applied the false discovery rate (FDR) correction on all receptors and found several

receptors showing statistically significant contributions on the gradient dynamics with arousal fluctuations (Fig. e). The above result is an example using the CHRM3 expression profile, and the procedure is summarized in Fig. e and applied to all neuromodulatory receptors.

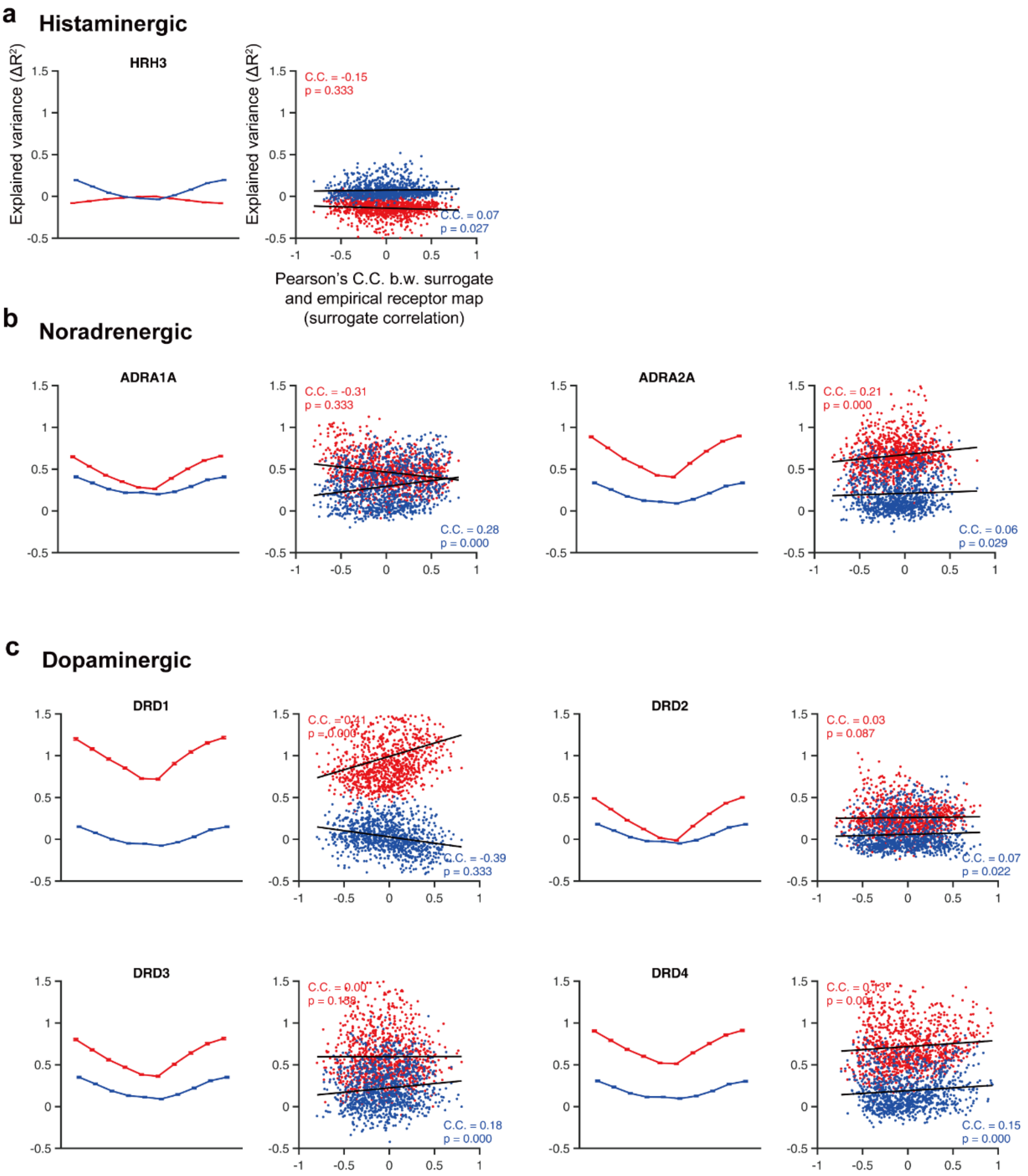

Supplementary Fig. 26 Statistical evaluation of the unique contribution of histaminergic (a), noradrenergic (b), dopaminergic (c), cholinergic (d) and serotonergic (e) receptors using the criterion in Supplementary Fig.25.

(Left panel) Arousal relevant dynamics of the unique contribution for a given receptor, showing an inverted U shape relationship using SA preserving surrogate maps as shuffling. Errorbar, Mean+/-SEM, n=1000 times shuffling.

(Right panel) Pearson's correlation coefficients (right-tailed *t*-test) between surrogate correlation and the variance of the unique contribution of a given receptor. If the correlation is not significant, it means random receptor maps could contribute similar arousal modulation, i.e., the empirical receptor does not specifically contribute to arousal dynamics. Alternatively, if the correlation is significant, it means larger spatial map shuffling causes larger loss of the unique contribution for the corresponding receptor, i.e., the empirical receptor does contribute to arousal dynamics. Each dot represented a random SA preserving shuffling of corresponding receptor (n = 1000 times).

(Supplementary Fig. 26, to be continue…, next page)

d **Cholinergic**

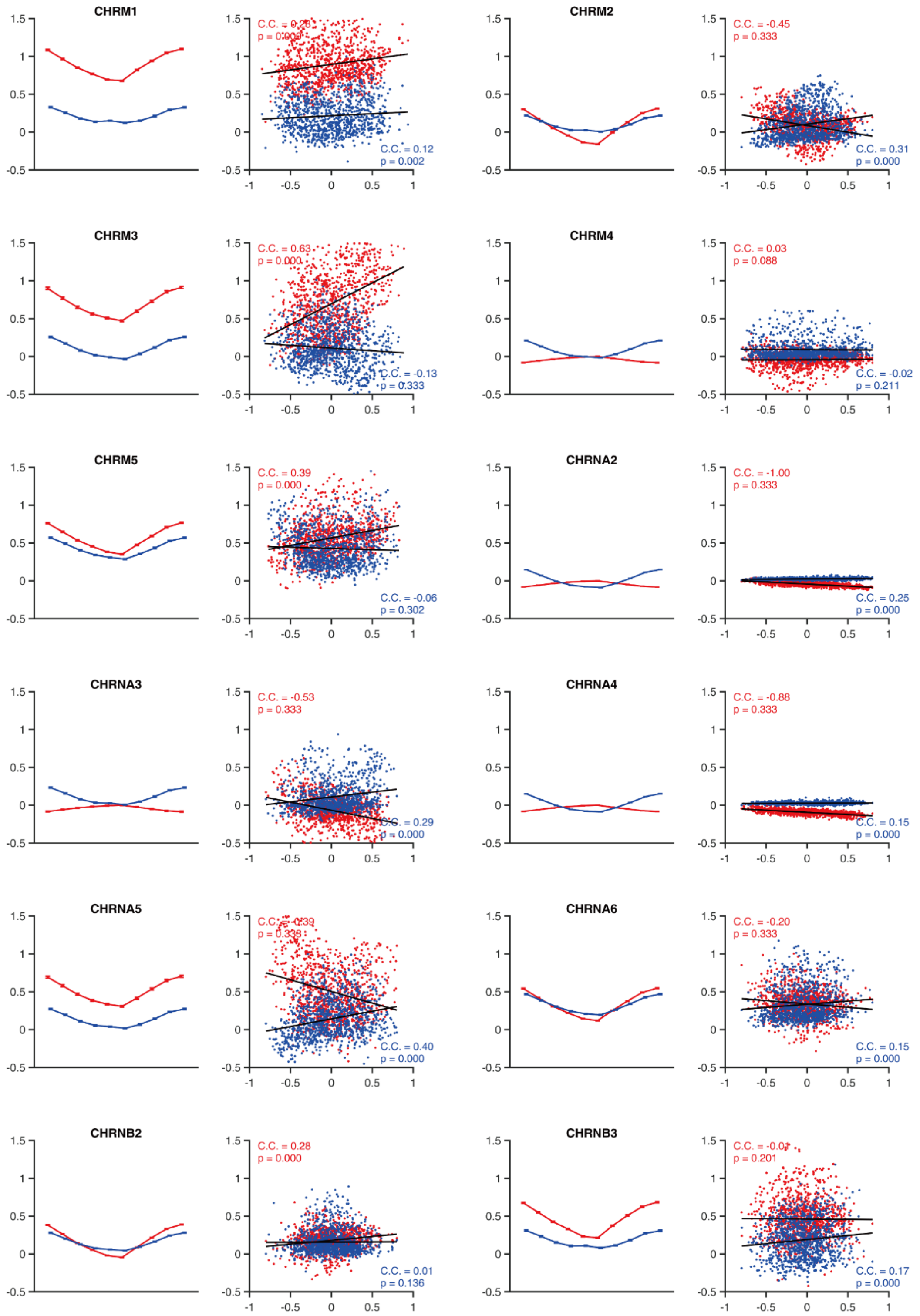

(Supplementary Fig. 26, to be continue…, next page)

e **Serotonergic**

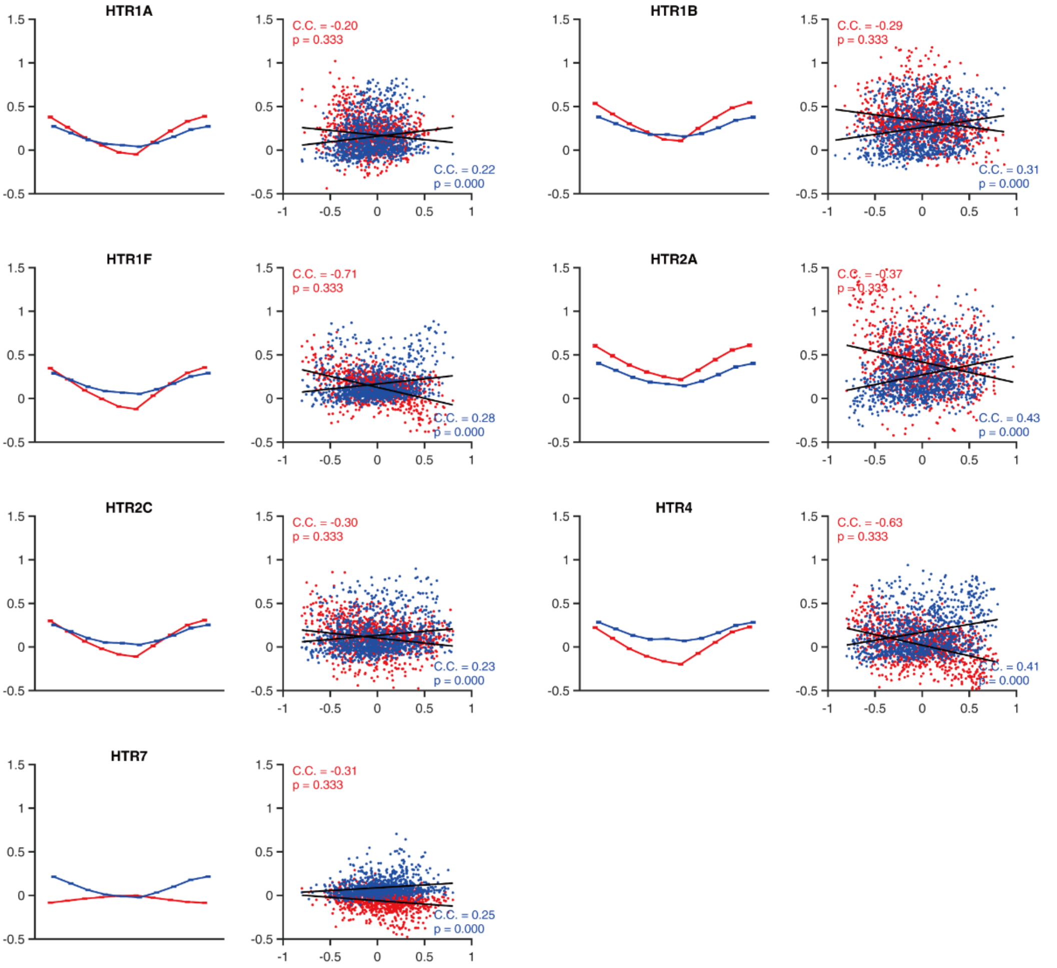

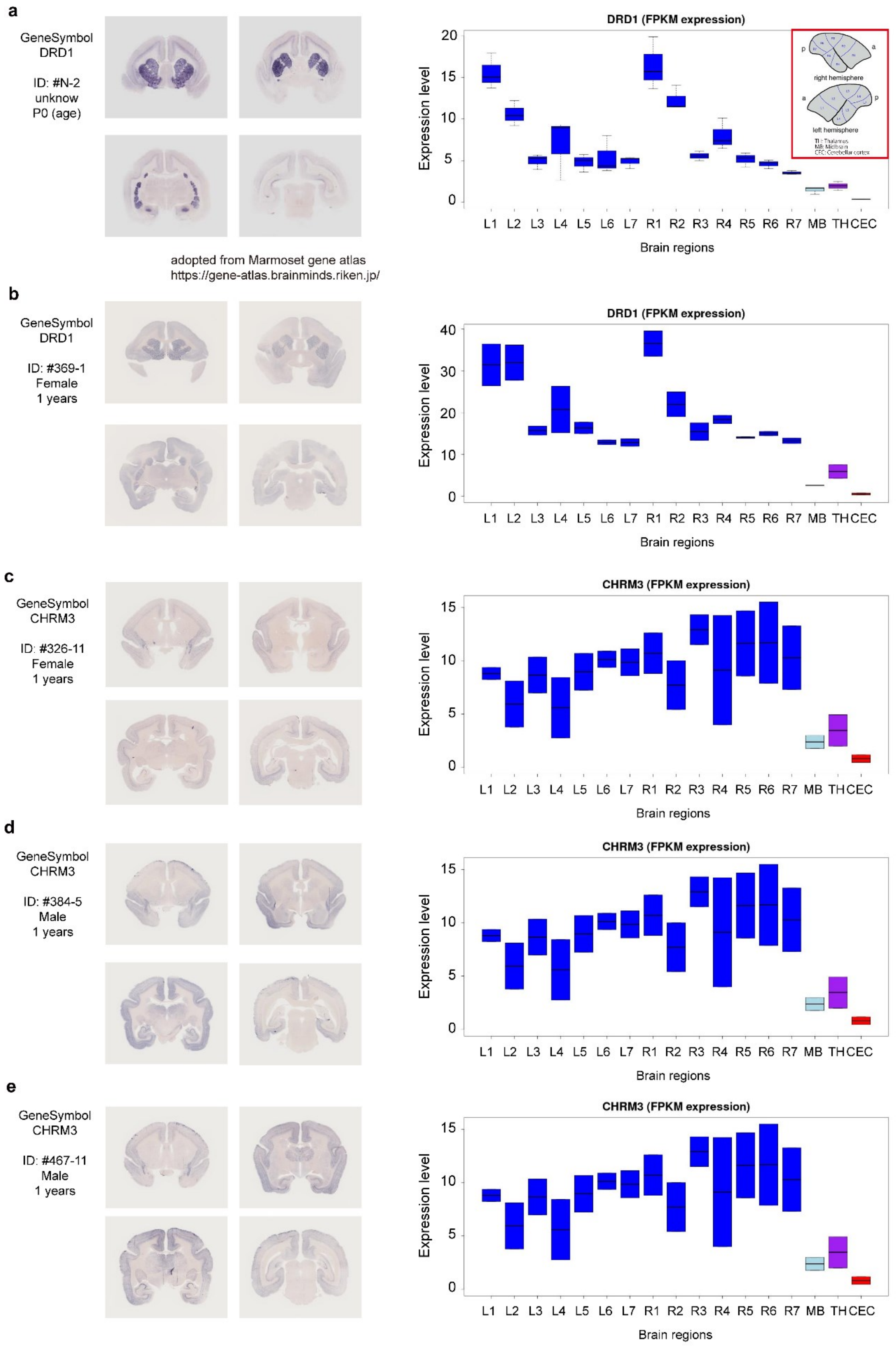

Supplementary Fig. 27 Consistency of marmoset gene expression information across sex, age and subjects.

(a-b) Expression pattern of marmoset dopamine receptor D1 (DRD1) from P0 (a) and 1

year (b) old marmosets. Left, example of marmoset gene expression images in coronal view. Right, quantitative evaluation of marmoset gene expression profiles. Insert, definitions of marmoset cortical parcellations used in the right panel. a, anterior; p, posterior. The parcellations and quantifications of expression levels were directly obtained from marmoset gene atlas database (https://gene-atlas.brainminds.riken.jp/).

On each box plot, the central mark indicates the mean, and the bottom and top edges of the box indicate the 25th and 75th percentiles, respectively. The whiskers extend to the most extreme data points not considered outliers.

On each floating bar plot, the central mark indicates the mean, and the bottom and top edges of the box indicate the minimal and maximal values.

Sample size for each box plot or floating bar plot was the number of pixels within corresponding cortical parcels.

(c-e) Similar to (a-b) but for cholinergic receptor muscarinic 3 (CHRM3) from a female 1 year old marmoset (c) and two male 1year old marmosets (d-e).

Supplementary Table 1. Abbreviations of marmoset brain networks.

<table>
<tr><th>Abbreviations</th><th>Voxels</th><th colspan="2">Full form</th></tr>
<tr><td>visual 0</td><td>100632</td><td rowspan="5">Visual network</td><td rowspan="5">Primary
↓
High order</td></tr>
<tr><td>visual 1</td><td>87573</td></tr>
<tr><td>visual 2</td><td>89933</td></tr>
<tr><td>visual 3</td><td>71297</td></tr>
<tr><td>visual 4</td><td>87664</td></tr>
<tr><td>AUD</td><td>74384</td><td colspan="2">Auditory network</td></tr>
<tr><td>dorsalsoma</td><td>73321</td><td colspan="2">Dorsal sensorimotor network</td></tr>
<tr><td>ventralsoma</td><td>51721</td><td colspan="2">Ventral sensorimotor network</td></tr>
<tr><td>premotor</td><td>69985</td><td colspan="2">Premotor network</td></tr>
<tr><td>parahip</td><td>77300</td><td colspan="2">Parahippocampus / temporal pole network</td></tr>
<tr><td>frontalpole</td><td>67239</td><td colspan="2">Frontal pole</td></tr>
<tr><td>OFC</td><td>46618</td><td colspan="2">Orbital frontal cortex</td></tr>
<tr><td>frontalpaietal</td><td>84491</td><td colspan="2">Frontal parietal network</td></tr>
<tr><td>DMN</td><td>97722</td><td colspan="2">Default mode network</td></tr>
<tr><td>ACC</td><td>60310</td><td colspan="2">Anterior cingulate / salience-like network</td></tr>
</table>

Supplementary Table 2. Genes of marmoset neuromodulatory receptors used in GLM and reduced model.

ID	GeneSymbol	GeneName	Sex	Age
#59-4	ADRA1A	adrenoceptor alpha 1A	F	P0
#163-2	ADRA2A	adrenoceptor alpha 2A	M	P0
#163-4	CHRM1	cholinergic receptor muscarinic 1	M	P0
#45-4	CHRM2	cholinergic receptor muscarinic 2	M	P0
#326-11	CHRM3	cholinergic receptor muscarinic 3	F	1Y
#318-5	CHRM4	cholinergic receptor muscarinic 4	M	P0
#47-4	CHRM5	cholinergic receptor muscarinic 5	M	P0
#240-5	CHRNA2	cholinergic receptor nicotinic alpha 2 subunit	F	P0
#313-2	CHRNA3	cholinergic receptor nicotinic alpha 3 subunit	F	P0
#109-8	CHRNA4	cholinergic receptor nicotinic alpha 4 subunit	M	P0
#136-1	CHRNA5	cholinergic receptor nicotinic alpha 5 subunit	M	P0
#37-7	CHRNA6	cholinergic receptor nicotinic alpha 6 subunit	F	P0
#183-1	CHRNB2	cholinergic receptor nicotinic beta 2 subunit	M	P0
#312-4	CHRNB3	cholinergic receptor nicotinic beta 3 subunit	F	P0
#369-1	DRD1	dopamine receptor D1	F	1Y
#AA-2	DRD2	dopamine receptor D2	-	P1
#5-3	DRD3	dopamine receptor D3	M	P0
#296-4	DRD4	dopamine receptor D4	M	P0
#270-8	HRH3	histamine receptor H3	F	P0
#84-2	HTR1A	5-hydroxytryptamine receptor 1A	F	P0
#104-2	HTR1B	5-hydroxytryptamine receptor 1B	M	P0
#62-8	HTR1F	5-hydroxytryptamine receptor 1F	F	P0
#78-7	HTR2A	5-hydroxytryptamine receptor 2A	M	P0
#84-4	HTR2C	5-hydroxytryptamine receptor 2C	F	P0
#184-1	HTR4	5-hydroxytryptamine receptor 4	F	P0
#323-6	HTR7	5-hydroxytryptamine receptor 7	F	P0
#AY-6	HTR7	5-hydroxytryptamine receptor 7	-	P0

Supplementary Table 3. Genes of marmoset glutamate receptors used in GLM and reduced model.

ID	GeneSymbol	GeneName	Sex	Age
#157-8	GRIA1	glutamate ionotropic receptor AMPA type subunit 1	F	P0
#261-2	GRIA2	glutamate ionotropic receptor AMPA type subunit 2	F	P0
#266-2	GRIA3	glutamate ionotropic receptor AMPA type subunit 3	M	P0
#160-2	GRIA4	glutamate ionotropic receptor AMPA type subunit 4	M	P0
#270-6	GRID1	glutamate ionotropic receptor delta type subunit 1	F	P0
#55-2	GRID2	glutamate ionotropic receptor delta type subunit 2	M	P0
#43-2	GRIK1	glutamate ionotropic receptor kainate type subunit 1	F	P0
#269-1	GRIK2	glutamate ionotropic receptor kainate type subunit 2	F	P0
#136-3	GRIK3	glutamate ionotropic receptor kainate type subunit 3	M	P0
#341-8	GRIK4	glutamate ionotropic receptor kainate type subunit 4	M	P0
#350-4	GRIK5	glutamate ionotropic receptor kainate type subunit 5	M	P0
#100-8	GRIN1	glutamate ionotropic receptor NMDA type subunit 1	M	P0
#109-2	GRIN2A	glutamate ionotropic receptor NMDA type subunit 2A	M	P0
#65-9	GRIN2B	glutamate ionotropic receptor NMDA type subunit 2B	F	3M
#270-5	GRIN3A	glutamate ionotropic receptor NMDA type subunit 3A	F	P0
#117-5	GRIN3B	glutamate receptor ionotropic, NMDA 3B	F	P0
#331-4	GRM1	glutamate metabotropic receptor 1	F	6M
#61-3	GRM2	glutamate metabotropic receptor 2	F	P0
#76-2	GRM3	glutamate metabotropic receptor 3	M	P0
#55-3	GRM4	glutamate metabotropic receptor 4-like	M	P0
#61-4	GRM6	glutamate metabotropic receptor 6	F	P0
#55-4	GRM7	glutamate metabotropic receptor 7	M	P0
#163-7	GRM8	glutamate metabotropic receptor 8	M	P0

Supplementary Table 4. Random selected marmoset genes used in GLM and reduced model.

ID	GeneSymbol	GeneName	Sex	Age
#64-4	ABI3BP	ABI family member 3 binding protein	F	3M
#331-12	ATP6V1C1	ATPase H+ transporting V1 subunit C1	F	6M
#265-9	BTBD3	BTB domain containing 3	F	6M
#31-8	CADPS2	calcium dependent secretion activator 2	M	3M
#64-7	CDH6	cadherin 6	F	3M
#31-7	CPLX3	complexin 3	M	3M
#31-5	ETV1	ETS variant transcription factor 1	M	3M
#31-11	FEZF2	FEZ family zinc finger 2	M	3M
#31-10	GABRQ	GABRQ gamma-aminobutyric acid type A receptor subunit theta	M	3M
#32-8	GAD1	glutamate decarboxylase 1	M	3M
#32-5	GBX2	gastrulation brain homeobox 2	M	3M
#31-6	GLRA3	glycine receptor alpha 3	M	3M
#230-6	GPR151	G protein-coupled receptor 151	F	6M
#230-11	HCRTR1	hypocretin receptor 1	F	6M
#265-1	HCRTR2	hypocretin receptor 2	F	6M
#331-11	ITPR1	inositol 1,4,5-trisphosphate receptor type 1	F	6M
#64-2	KITLG	KIT ligand	F	3M
#31-9	MET	MET proto-oncogene, receptor tyrosine kinase	M	3M
#230-7	MTNR1A	melatonin receptor 1A	F	6M
#64-1	NR1D1	nuclear receptor subfamily 1 group D member 1	F	3M
#31-3	NR4A2	nuclear receptor subfamily 4 group A member 2	M	3M
#32-10	NTNG1	netrin G1	M	3M
#230-9	POU3F4	POU class 3 homeobox 4	F	6M
#32-11	PRKCD	protein kinase C delta	M	3M
#32-1	PVALB	parvalbumin	M	3M
#151-5	RORA	RAR related orphan receptor A	F	3M
#31-1	SATB2	SATB homeobox 2	M	3M
#331-10	SCN1B	sodium voltage-gated channel beta subunit 1	F	6M
#32-7	SLC17A7	solute carrier family 17 member 7	M	3M
#32-6	SLC18A3	solute carrier family 18 member A3	M	3M
#25-8	SLC6A3	solute carrier family 6 member 3	F	4M
#230-5	SNX31	sorting nexin 31	F	6M
#230-8	SOX6	SRY-box transcription factor 6	F	6M
#230-10	WNT7B	Wnt family member 7B	F	6M
#64-5	ZIC4	Zic family member 4	F	3M

Supplementary References

1 Burt, J. B., Helmer, M., Shinn, M., Anticevic, A. & Murray, J. D. Generative modeling of brain maps with spatial autocorrelation. *Neuroimage* **220**, 117038, (2020).

2 Markello, R. D. & Misic, B. Comparing spatial null models for brain maps. *Neuroimage* **236**, 118052, (2021).

3 Demirtas, M. *et al.* Hierarchical Heterogeneity across Human Cortex Shapes Large-Scale Neural Dynamics. *Neuron* **101**, 1181-1194 e1113, (2019).
