# Supplementary material for: Multimodal analysis demonstrating the shaping of functional gradients in the marmoset brain
Source: Nat Commun. 2022 Nov 3;13:6584. doi: 10.1038/s41467-022-34371-w (PMC9633775; doi:10.1038/s41467-022-34371-w)
Supplement: Supplementary file 3 — Reporting Summary [file 41467_2022_34371_MOESM3_ESM.pdf]

nature portfolio

Corresponding author(s): Zhifeng Liang

Last updated by author(s): Oct 17, 2022

Reporting Summary

Nature Portfolio wishes to improve the reproducibility of the work that we publish. This form provides structure for consistency and transparency in reporting. For further information on Nature Portfolio policies, see our Editorial Policies and the Editorial Policy Checklist.

Statistics

For all statistical analyses, confirm that the following items are present in the figure legend, table legend, main text, or Methods section.

n/a	Confirmed	
☐	☒	The exact sample size (*n*) for each experimental group/condition, given as a discrete number and unit of measurement
☐	☒	A statement on whether measurements were taken from distinct samples or whether the same sample was measured repeatedly
☐	☒	The statistical test(s) used AND whether they are one- or two-sided<br>*Only common tests should be described solely by name; describe more complex techniques in the Methods section.*
☐	☒	A description of all covariates tested
☐	☒	A description of any assumptions or corrections, such as tests of normality and adjustment for multiple comparisons
☐	☒	A full description of the statistical parameters including central tendency (e.g. means) or other basic estimates (e.g. regression coefficient) AND variation (e.g. standard deviation) or associated estimates of uncertainty (e.g. confidence intervals)
☐	☒	For null hypothesis testing, the test statistic (e.g. *F*, *t*, *r*) with confidence intervals, effect sizes, degrees of freedom and *P* value noted<br>*Give P values as exact values whenever suitable.*
☒	☐	For Bayesian analysis, information on the choice of priors and Markov chain Monte Carlo settings
☒	☐	For hierarchical and complex designs, identification of the appropriate level for tests and full reporting of outcomes
☐	☒	Estimates of effect sizes (e.g. Cohen's *d*, Pearson's *r*), indicating how they were calculated

Our web collection on statistics for biologists contains articles on many of the points above.

Software and code

Policy information about availability of computer code

Data collection	ParaVision Version 6.0.1 for MRI acquisitions
Data analysis	ITK-SNAP (http://www.itksnap.org/) for marmoset brain extraction; MATLAB 2020a (MathWorks, Natick, MA) and SPM12 (http://www.fil.ion.ucl.ac.uk/spm/) for fMRI processing; FSL (FMRIB Software Library v5.1, Oxford University, Oxford UK) for EPI distortion correction (topup); Freesurfer version 5.2 for human fMRI coregistration; HCP Connectome Workbench version 1.5.0 for human fMRI surface rendering; BrainSpace toolbox (Vos de Wael, R., et al., Commun Biol, 2020) for the computation of macroscale gradients; DCC toolbox (Lindquist MA, et al., Neuroimage, 2014) for dynamic conditional correlation analysis; Graphpad Prism 8(https:// www.graphpad.com/) for data plot.

For manuscripts utilizing custom algorithms or software that are central to the research but not yet described in published literature, software must be made available to editors and reviewers. We strongly encourage code deposition in a community repository (e.g. GitHub). See the Nature Portfolio guidelines for submitting code & software for further information.

Data

Policy information about availability of data

All manuscripts must include a data availability statement. This statement should provide the following information, where applicable:

- Accession codes, unique identifiers, or web links for publicly available datasets
- A description of any restrictions on data availability
- For clinical datasets or third party data, please ensure that the statement adheres to our policy

The manuscript released a dual center resting-state functional MRI dataset, which is open public available via Marmoset Brain Mapping Resource website (https://marmosetbrainmapping.org). The raw resting-state MRI data are provided in the standard BIDS format for cross-platform sharing. The marmoset neuro-tracing data

(https://www.marmosetbrain.org/) and gene expression data (https://gene-atlas.brainminds.riken.jp/) was published previously as open resource. All human fMRI data are free provided by the Human Connectome Project (https://www.humanconnectome.org/).

Field-specific reporting

Please select the one below that is the best fit for your research. If you are not sure, read the appropriate sections before making your selection.

☒ Life sciences ☐ Behavioural & social sciences ☐ Ecological, evolutionary & environmental sciences

For a reference copy of the document with all sections, see nature.com/documents/nr-reporting-summary-flat.pdf

Life sciences study design

All studies must disclose on these points even when the disclosure is negative.

Sample size	No sample-size calculation was performed.<br>Generally for resting-state fMRI, large data is preferred. The current study utilized the largest awake marmoset resting-state fMRI data so far, including 39 marmosets and a total of 709 runs and 12053 min data (many marmosets were repeatedly imaged).<br>This sample size have shown to be sufficient to investigate the properties of functional gradients (e.g. 637 human participants with 637 EPI runs, Richard A.I. Bethlehem, Casey Paquola, et. al., Neuroimage, 2020; 378 human participants with 756 EPI runs, Dong., et. al., PNAS, 2021).<br>Also, the sample size is adequate to address our scientific questions, because the population-level functional gradients produced highly similar results between ION and NIH datasets.
Data exclusions	For each fMRI run, any runs were excluded if the maximum head motion was > 0.2 mm.
Replication	n/a<br>For resting-state fMRI data collection, identical imaging procedures were applied to different marmosets or humans,which formed the imaging dataset in this manuscript. Thus, the conventional concept of biological replication was not applicable.<br>For the replication of scientific insights, we replicated same analysis using the ION and NIH dataset (Fig.1), showing high cross-dataset similarity between ION and NIH datasets.
Randomization	n/a<br>There are no experimental and control groups in our study. All marmoset and human resting state fMRI data were single groups.
Blinding	n/a<br>There are no experimental and control groups in our study. All marmoset and human resting state fMRI data were single groups.<br>Blinding was not necessary, since all marmoset or human individuals were passed through identical processing pipelines.

Reporting for specific materials, systems and methods

We require information from authors about some types of materials, experimental systems and methods used in many studies. Here, indicate whether each material, system or method listed is relevant to your study. If you are not sure if a list item applies to your research, read the appropriate section before selecting a response.

Materials & experimental systems

n/a	Involved in the study	
☒	☐	Antibodies
☒	☐	Eukaryotic cell lines
☒	☐	Palaeontology and archaeology
☐	☒	Animals and other organisms
☐	☒	Human research participants
☒	☐	Clinical data
☒	☐	Dual use research of concern

Methods

n/a	Involved in the study	
☒	☐	ChIP-seq
☒	☐	Flow cytometry
☐	☒	MRI-based neuroimaging

Animals and other organisms

Policy information about studies involving animals; ARRIVE guidelines recommended for reporting animal research

Laboratory animals	Marmoset (Callithrix jacchus); ION: 13 males and 1 female (3 +/- 1 years old); NIH: 19 males and 7 females (4 +/- 2 years old).
Wild animals	This study did not involve wild animals.
Field-collected samples	The study did not involve samples collected from the field.
Ethics oversight	The experimental procedures were approved by the Animal Care and Use Committees from the Institute of Neuroscience (ION) at

Ethics oversight | the Chinese Academy of Sciences and National Institute of Neurological Disorders and Stroke at the National Institutes of Health (NIH).

Note that full information on the approval of the study protocol must also be provided in the manuscript.

Human research participants

Policy information about studies involving human research participants

Population characteristics | Age = 29.2 ± 3.5, range: 22~36, 194 males and 275 females.

Recruitment | Subject recruitment procedures and informed consent forms, including consent to share de-identified data, were approved by the Washington University institutional review board.

Ethics oversight | Institutional Review Board at Washington University in St. Louis.

Note that full information on the approval of the study protocol must also be provided in the manuscript.

Magnetic resonance imaging

Experimental design

Design type | resting state (marmoset and human)

Design specifications | Functional MRI were collected at resting state for both marmoset and human.

Behavioral performance measures | For ION marmoset dataset, respiratory and cardiac signals, as well as eye-monitoring videos, were recorded.

Acquisition

Imaging type(s) | Marmoset: Functional (resting state fMRI); Structural (T2-weighed anatomical image)
Human: Functional (resting state fMRI); Structural (T1- and T2-weighed anatomical image)

Field strength | Marmoset: (ION) 9.4T, Bruker; (NIH) 7T, Bruker
Human: 3T, Siemens

Sequence & imaging parameters | Marmoset: For each session, multiple runs of rsfMRI data were collected using 2D gradient echo EPI sequence with the following parameters: TR = 2000 ms, TE = 18 ms (ION) or 22.2 ms (NIH), flip angle = 70.4°, FOV = 28 ×36 mm, matrix size = 56 × 72, 38 axial slices, slice thickness = 0.5 mm, 512 volumes per scan. Two sets of spin-echo EPI with opposite phase-encoding directions (LR and RL) were also collected for EPI-distortion correction with following parameters: TR= 3000ms, TE=37.69 ms (ION) or 36 ms (NIH), flip angle=90°, FOV=28 ×36 mm, matrix size=56×72, 38 axial slices, slice thickness=0.5 mm, 8 volumes for each set. After each rsfMRI session, a T2-weighted structural image was acquired for co-registration with following parameters: TR=8000 ms (ION) or 6000 ms (NIH), TE=10 ms (ION) or 9 ms (NIH), flip angle=90°, FOV= 28 ×36mm, matrix size=112 ×144, 38 axial slices, slice thickness=0.5mm.

Human: (1) Gradient echo EPI : TR = 720 ms, using a multiband factor of 8, FA = 52°,2 mm isotropic spatial resolution. FOV: 208mm × 180mm, Matrix: 104 × 90 with 72 slices covering the entire brain. (2) T1-w anatomical image: 0.7mm isotropic resolution (FOV=224 mm, matrix=320, 256 sagittal slices in a single slab), TR=2400 ms, TE=2.14 ms, TI=1000 ms, FA=8°, Bandwidth (BW)=210 Hz per pixel, Echo Spacing (ES) =7.6 ms, with a non-selective binomial (1:1) water excitation pulse (a pair of 100 µs hard pulses with 1.2 ms spacing) to reduce signal from bone marrow and scalp fat, phase encoding undersampling factor GRAPPA=2. (3) T2-w anatomical image: 0.7mm isotropic resolution (same matrix, FOV, and slices as in the T1w), TR=3200 ms, TE=565 ms, BW=744 Hz per pixel, no fat suppression pulse, phase encoding undersampling factor GRAPPA=2, total turbo factor=314.
The acquisition parameters of human fMRI were described in details by the HCP consortium.

Area of acquisition | Whole brain

Diffusion MRI ☐ Used ☒ Not used

Preprocessing

Preprocessing software | Marmoset: ITK-SNAP for marmoset brain extraction; MATLAB 2020a and SPM12 for fMRI processing; FSL for EPI distortion correction (topup); After distortion correction, EPI images were realigned first for motion correction, and coregistered to the anatomical template. A light spatial smoothing (0.5 mm FWHM isotropic) and a band-pass filter (0.005~0.1 Hz) were also performed.
Human: Preprocessing was performed by the HCP consortium using ICA-FIX approach. Packages were FSL 5.0.6, Freesurfer 5.3.0-HCP, Connectome Workbench V1.1.1. In addition to the preprocessing steps implemented by the HCP, we applied smoothing both spatially (Gaussian filter with the FWHM = 2.4 mm) and temporally (band-pass filtered at 0.005–0.1 Hz).

Normalization | The fMRI data was coregistered to the subject's own T2 anatomical images (spm12, coregistration), which were further coregistered to a brain template (spm12, oldnormalize).

Normalization template	Marmoset: MRM v3.0 template.<br>Human: ICBM 152 template.
Noise and artifact removal	Marmoset: The resting state fMRI data were further regressed by 22 “nuisance signals” to reduce motion artifacts, including 6 head motion parameters, their 1st order derivatives and 10 non-brain tissue based principal components (PCs). We also conducted parallel analyses on data with ICA-FIX de-noising and obtained very similar results.<br>Human: The resting-state fMRI data were further denoised using the ICA-FIX method.
Volume censoring	No fMRI volume was censored during fMRI preprocessing.

Statistical modeling & inference

Model type and settings	Not relevant to this study
Effect(s) tested	Not relevant to this study

Specify type of analysis: ☐ Whole brain ☐ ROI-based ☒ Both

Anatomical location(s)	Regions of interest (ROIs) were defined on previous MRI atlas (Marmoset: MRM v3.0, https://marmosetbrainmapping.org/atlas.html; Human: Schaefer. et al, 2018).
Statistic type for inference (See Eklund et al. 2016)	Not relevant to this study
Correction	Not relevant to this study

Models & analysis

n/a	Involved in the study
☐	☒ Functional and/or effective connectivity
☒	☐ Graph analysis
☐	☒ Multivariate modeling or predictive analysis

Functional and/or effective connectivity	Functional connectivity was measured as inter-regional correlations (Pearson correlation).
Multivariate modeling and predictive analysis	We constructed three GLM models (full model, single variable model and reduced model) to dissect the contribution on functional gradients from structural connectivity and neuromodulatory similarity. For the full model, all variables were included in the GLM model. The single variable model only included one variable in the GLM model. However, we concluded that the two predictors shared large overlap, evidenced by high correlation between predicted gradients from two single variable models. To address this issue, we constructed the reduced model to capture the unique contribution of each variables by applying random shuffling to a particular variable (1000 times). The resulting loss of explained variance captured the unique contribution of the corresponding variable. The single variable model provided an upper bound for the given variable, while the reduced model provided a lower bound for the unique contribution of the corresponding variable.
